# In Depth Characterization of Repetitive DNA in 23 Plant Genomes Reveals Sources of Genome Size Variation in the Legume Tribe *Fabeae*

**DOI:** 10.1371/journal.pone.0143424

**Published:** 2015-11-25

**Authors:** Jiří Macas, Petr Novák, Jaume Pellicer, Jana Čížková, Andrea Koblížková, Pavel Neumann, Iva Fuková, Jaroslav Doležel, Laura J. Kelly, Ilia J. Leitch

**Affiliations:** 1 Biology Centre of the Czech Academy of Sciences, Institute of Plant Molecular Biology, České Budějovice, Czech Republic; 2 Jodrell Laboratory, Royal Botanic Gardens, Kew, Richmond, Surrey, United Kingdom; 3 Institute of Experimental Botany, Olomouc, Centre of the Region Haná for Biotechnological and Agricultural Research, Olomouc, Czech Republic; 4 School of Biological and Chemical Sciences, Queen Mary University of London, London, United Kingdom; Leibniz-Institute of Plant Genetics and Crop Plant Research (IPK), GERMANY

## Abstract

The differential accumulation and elimination of repetitive DNA are key drivers of genome size variation in flowering plants, yet there have been few studies which have analysed how different types of repeats in related species contribute to genome size evolution within a phylogenetic context. This question is addressed here by conducting large-scale comparative analysis of repeats in 23 species from four genera of the monophyletic legume tribe *Fabeae*, representing a 7.6-fold variation in genome size. Phylogenetic analysis and genome size reconstruction revealed that this diversity arose from genome size expansions and contractions in different lineages during the evolution of *Fabeae*. Employing a combination of low-pass genome sequencing with novel bioinformatic approaches resulted in identification and quantification of repeats making up 55–83% of the investigated genomes. In turn, this enabled an analysis of how each major repeat type contributed to the genome size variation encountered. Differential accumulation of repetitive DNA was found to account for 85% of the genome size differences between the species, and most (57%) of this variation was found to be driven by a single lineage of Ty3/gypsy LTR-retrotransposons, the Ogre elements. Although the amounts of several other lineages of LTR-retrotransposons and the total amount of satellite DNA were also positively correlated with genome size, their contributions to genome size variation were much smaller (up to 6%). Repeat analysis within a phylogenetic framework also revealed profound differences in the extent of sequence conservation between different repeat types across *Fabeae*. In addition to these findings, the study has provided a proof of concept for the approach combining recent developments in sequencing and bioinformatics to perform comparative analyses of repetitive DNAs in a large number of non-model species without the need to assemble their genomes.

## Introduction

The discrepancy between the amount of DNA in a non-replicated haploid nucleus (C-value) and the complexity of eukaryotic organisms, also known as the C-value paradox [1], has long puzzled geneticists and evolutionary biologists. Multiple lines of research, starting with the pioneering works employing DNA reassociation kinetics [2,3] and culminating in the recent application of high throughput genome sequencing technologies have provided evidence that genome size variation is primarily driven by the differential accumulation and elimination of repetitive DNA, whereas the number of genes remains relatively stable [4]. These findings have led to the proposal of an alternative term, the C-value enigma, reflecting the fact that although there is now no paradox in the causes of the observed genome size variation, there is still relatively little known about how the various molecular and evolutionary mechanisms contribute to genome size diversification in different groups of organisms [5,6].

The repetitive fractions of eukaryotic genomes are very complex. They include diverse families of dispersed mobile elements and tandemly organized satellite repeats, with the relative proportions and sequence compositions of these repeats differing considerably between taxa. In spite of the selfish nature of most repetitive elements, there are an increasing number of examples showing that some repeats are beneficial or even essential for a genome [4], highlighting the diverse ways that repeats can impact on genome organization, function and evolution [7]. To explain the differential accumulation of repetitive DNA observed in eukaryotic genomes, several contrasting explanations have been proposed, ranging from it being a passive consequence of non-adaptive processes [8] to considering repeat amplification as an initial step and major prerequisite for evolutionary radiation [9]. Consequently, there is a need for a thorough characterization of repeats at various scales, from individuals and species to higher taxa, in order to test the validity of the proposed hypotheses or to develop new ones [10].

Flowering plants (angiosperms) are amongst the best models to study the impact of repetitive DNA on genome evolution as they exhibit extraordinary variation in genome size, spanning over three orders of magnitude (from c. 0.59 Gbp/1C in the carnivorous plant *Genlisea tuberosa* [11] to 148.90 Gbp/1C in *Paris japonica* [12]). Although satellite repeats can account for as much as 10–20% of the genome in some species [13,14], the bulk of repeats are usually made up of mobile elements [15–19]. Of these, it has been shown that LTR-retrotransposons represent the major repeat fraction in most plant genomes and their accumulation is, along with multiple rounds of polyploidization, a key force governing genome size expansion [20,21]. Nevertheless, repeats can also be removed from the genome by the action of various recombination-based mechanisms [22] leading to reductions in genome size [23,24]. It has been suggested that the relative efficiency of these opposing forces sets the trend of genome size evolution [14], however it is still unclear what determines this balance in different taxa, leading to the huge variation of genome sizes found in extant species [25].

One way to gain a comprehensive understanding of the processes shaping the repeat composition of plant genomes is to conduct a detailed characterization of repetitive DNA in multiple species followed by analysis within a phylogenetic framework. Until recently, such studies were scarce due to the large amounts and considerable sequence complexity of plant repeats that would need to be analysed. However, much progress has been made in recent years due to the introduction of next generation sequencing (NGS) technologies and corresponding bioinformatic approaches (reviewed in [26]). One of these novel approaches, the similarity-based clustering of low coverage genome sequencing reads [19], has proved to be particularly efficient for repeat identification and characterization in eukaryotic genomes [16–18,24,27–30]. It conducts all-to-all pairwise comparison of the reads and groups those reads which share significant sequence similarities into ‘clusters’. These clusters mostly represent repeats, because only the reads derived from sequences present in the genome multiple times can produce a sufficient number of similarity hits in the low-pass sequencing data (0.01–0.50x genome coverage is typically used). In principle, the number of reads in each cluster is proportional to the genomic abundance of the corresponding repeat, thus enabling its quantification. In the most recent implementation of this approach, the identification and characterization of repeat clusters has been enhanced by representing the reads and their sequence similarities as nodes and connecting edges, respectively, in a virtual graph, and examination of the graph topology [31]. The graph-based read clustering has become the core algorithm for *RepeatExplorer*, a computational pipeline designed for identification, quantification and annotation of repeats in plant genomes [32].

In the present work, we have extended this bioinformatic approach by introducing several novel methods for repeat characterization and applied them to analyse the genomes of 23 species belonging to the legume tribe *Fabeae*. This tribe includes four main genera, *Vicia*, *Lathyrus*, *Pisum* and *Lens* and was selected for this study because of the considerable diversity of genome sizes reported to exist (1.8–14.3 Gbp/1C [33]) and the availability of a well resolved phylogenetic tree [34]. Moreover, previous results from just a few species belonging to this tribe have indicated that repetitive DNA has clearly played an important role in the evolution of their genomes [19,21,35–38]. Due to the large number of *Fabeae* species recognised (c. 380 species [34]), we have mainly focused our study on *Vicia*, which alone still covers a substantial part of the genome size diversity encountered in the tribe (1.8–13.4 Gbp/1C). By combining advanced sequencing and bioinformatic approaches, we have been able to characterize to an unprecedented depth the repeats that make up the majority of the nuclear genomes in all investigated species. In addition, the analysis of these data within an evolutionary framework has enabled us to gain novel insights into repeat dynamics across the *Fabeae*.

## Results

### Genome size diversification in the light of *Fabeae* phylogeny

The 23 *Fabeae* species selected for analysis are listed in Table 1. Since there was a relatively large discrepancy between previously reported genome size estimates for some species [33], we re-measured genome sizes for all accessions in this study using flow cytometry (S1 Table). Nevertheless, the results confirmed the expected genome size variation, ranging from 1.77 Gbp/1C in *V*. *sativa* to 13.41 Gbp/1C in *V*. *faba* (7.6-fold difference, Table 1). All accessions were diploid with chromosome numbers of 2n = 10, 12 or 14, except for *Vicia cracca* which was shown to be a tetraploid cytotype (2n = 4x = 28), most likely of autopolyploid origin [39]. Thus, in subsequent analyses we used monoploid genome size (1Cx) for *V*. *cracca*, which is comparable to the holoploid genome size (1C) in diploid species [40] (S1 Table).

**Table 1:** Estimation of genome size and sequencing of selected *Fabeae* species.

Species	Code	Accession		Genome size	Sequencing	Clustering max. reads		Clust. 0.01x
		Source^(^ ^1^ ^)^	Code	1Cx [Gbp]^(^ ^2^ ^)^	run acc.no.	reads	coverage	[reads]
***Vicia***								
*V*. *sativa* 'Ebena'	**VSA**	commercial		**1.77**	ERR413103	1050158	0.059	177360
*V*. *villosa*	**VVL**	IPK	VIC876	**2.04**	ERR413122	2671672	0.131	203620
*V*. *lathyroides* L.	**VLT**	IPK	VIC874	**2.43**	ERR413100	4812664	0.198	242544
*V*. *cracca* L. var. *cracca*	**VCR**	IPK	VIC71	**2.90**	ERR413096	6292470	0.217	289684
*V*. *tetrasperma* (L.) Schreb.	**VTS**	IPK	VIC726	**3.05**	ERR413111	7873852	0.258	305381
*V*. *sepium* L.	**VSP**	IPK	VIC55	**3.74**	ERR413104, ERR413105	1897538	0.051	374378
*V*. *grandiflora*	**VGR**	IPK	VIC741	**3.78**	ERR413106	3289836	0.087	378095
*V*. *hirsuta* (L.) S.F.Gray	**VHR**	IPK	VIC728	**3.88**	ERR413114	6606736	0.170	387533
*V*. *ervilia* (L.) Willd.	**VER**	IPK	ERV52	**4.06**	ERR413112	4593996	0.113	405723
*V*. *unijuga* A.Br.	**VUN**	IPK	VIC78	**4.37**	ERR413109	7201796	0.165	436775
*V*. *pannonica* 'Dětěnická panonská'	**VPN**	commercial		**5.73**	ERR413097, ERR413098	2101204	0.037	573108
*V*. *pisiformis* L.	**VPF**	IPK	VIC36	**6.15**	ERR413110	4460146	0.072	615407
*V*. *narbonensis*	**VNR**	ICARDA	14	**6.69**	ERR413121	3588026	0.054	668708
*V*. *sylvatica* L.	**VSL**	IPK	VIC63	**6.98**	ERR413113	4883944	0.070	698292
*V*. *melanops* Sibth. et Sm. var. *melanops*	**VML**	IPK	VIC474	**8.07**	ERR413101	3595696	0.045	806606
*V*. *peregrina* L.	**VPR**	IPK	VIC765	**8.45**	ERR413099	6197134	0.073	844650
*V*. *faba* 'Merkur'	**VFB**	commercial		**13.41**	ERR413107, ERR413108	3192982	0.024	1340985
***Lens***								
*L*. *culinaris* 'Eston'	**LNS**	commercial		**4.29**	ERR413115	5854630	0.137	428902
***Lathyrus***								
*L*. *vernus* (L.) Bernh.	**LAV**	natural population		**5.91**	ERR413116, ERR413117	6632676	0.112	591250
*L*. *sativus* L.	**LAS**	commercial		**6.52**	ERR413118, ERR413119	3308288	0.051	652473
*L*. *latifolius* L.	**LAL**	commercial		**9.98**	ERR413120	3091852	0.031	997756
***Pisum***								
*P*. *sativum* 'Terno'	**PST**	commercial		**4.36**	ERR063464	4525544	0.104	436237
*P*. *fulvum*	**PFL**	ICARDA	IG64207	**4.69**	ERR413083	5015824	0.107	468804

^(1)^ Seedbank abbreviations: *IPK*, Leibniz Institute for Plant Genetics and Crop Plant Research; *ICARDA*, International Center for Agricultural Research in the Dry Areas

^(2)^ In diploids (all species except for the tetraploid *V*. *cracca*) 1Cx = 1C. See S1 Table for details on genome size estimation.

**Fig 1:**
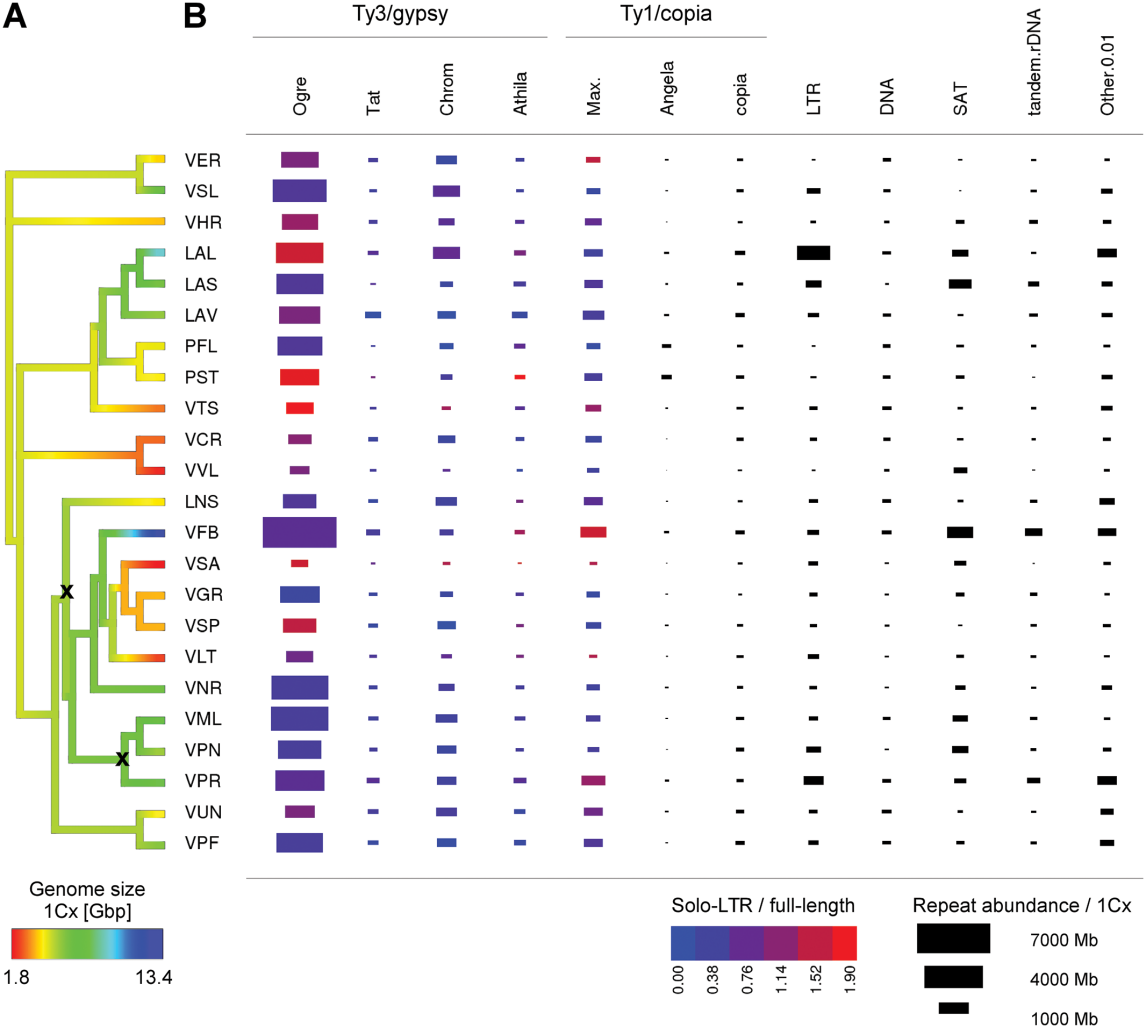
Genome size evolution and repeat composition of *Fabeae* species. (*A*) Phylogenetic tree of the 23 investigated species with their genome sizes shown by the colours of the terminal branches. Colour gradients within the tree branches indicate inferred genome size changes and species names are represented by the codes given in Table 1. All nodes except those labelled with "x" were highly supported with posterior probabilities >0.95 (see S1 Fig for details). (*B*) Graphical representation of the genomic abundances of major types of repetitive sequences. The area of the rectangles are proportional to the total length of individual repeats per monoploid genome size (1Cx) (see S2 Table). For LTR-retrotransposons, the colour of the rectangle indicates the estimated ratio of solo-LTRs to full-length elements (data given in Table 3). Repeat abbreviations: *Chrom*, Chromovirus; *Max*., Maximus/SIRE; *DNA*, DNA (class II) transposons; *SAT*, satellite repeats. *Copia* includes all Ty1/copia lineages except Maximus/SIRE and Angela; *LTR*, unclassified LTR-retrotransposons, *Other 0*.*01* includes remaining repeats with abundance exceeding 0.01% of the genome.

Phylogenetic relationships of the species studied here were evaluated using a set of molecular markers, including both rDNA (ITS) and plastid (*matK*, *trnS-G*) sequences. After inspection for conflicting signals between the markers, partitions were combined and analysed together, and the resulting phylogenetic tree is presented in Fig 1A. The evolutionary relationships of the species analysed are not fully resolved, but nevertheless they are largely consistent with those presented by Schaefer et al. [34] and show contrasting patterns of genome size evolution in different lineages. For example, the ancestral genome size reconstruction provides evidence for a trend towards genome expansion in *Lathyrus* species (LAL, LAS, LAV) while their closest relatives, *Pisum* (PST, PFL) and especially *V*. *tetrasperma* (VTS) show the opposite trend with respect to their most recent common ancestors (Fig 1A and S1 Fig). Genome size reduction is also a likely cause of diversification of smaller genomes within the group comprising *V*. *sativa/V*. *grandiflora/V*. *sepium/V*. *lathyroides* (VSA/VGR/VSP/VLT), and contrasts strikingly with that of their closest relative, *Vicia faba* (VFB), which has the largest genome so far reported for the tribe. Some pairs of closely related species differed considerably in genome size, including, for example, *V*. *ervilia* and *V*. *sylvatica* (VER, VSL, 1.72-fold/1C) and *V*. *unijuga* and *V*. *pisiformis* (VUN, VPF, 1.40-fold/1C). Overall these observations suggest that the genome size diversity encountered in the studied species is the result of bi-directional evolution taking place independently in several phylogenetic lineages of *Fabeae*.

### Repeat composition of *Fabeae* genomes

Whole genome shotgun sequencing was performed on all species using the Illumina platform generating 100 nt paired-end reads. Resulting sequences were analysed using the *RepeatExplorer* pipeline to identify clusters of frequently overlapping reads representing different repetitive elements or their parts [31,32]. The clustering analysis was performed for each species separately to maximize the number of analysed reads and hence the sensitivity and accuracy of the repeat data obtained for each species (the number of analysed reads and corresponding genome coverage are provided in the column "Clustering max. reads" of Table 1). Clusters representing repeats making up at least 0.01% of the genome were further characterized and quantified to provide detailed information about all types of moderately to highly abundant repetitive elements. The combined proportions of these repeat clusters varied from 59.5% in the smallest genome of *V*. *sativa* up to 85.7% in *V*. *sylvatica*. Most (91–99%) clusters were assigned to specific repeat types and families, resulting in the overall annotation of repeats representing 54.9–83.1% of the investigated genomes (S2 Table).

The global repeat composition of individual species is summarized in Fig 1B and listed in detail in S2 Table. To evaluate repeat content with respect to genome size differences between the species, we expressed estimated quantities of individual repeat types as their total length per monoploid genome (Mbp/1Cx—see Table A in S2 Table). An alternative expression in terms of genome proportion (%) is also provided (Table B in S2 Table).

Repeats classified as LTR-retrotransposons represented the major fraction of all analysed genomes, comprising up to 81% of their nuclear DNA (e.g. *V*. *sylvatica*). They were mostly represented by highly amplified and heterogeneous populations of Ogre elements, which alone constituted 7.3 Gbp (54%) of the largest *V*. *faba* genome and were the most abundant repeats in all species (Fig 1B). Ogres belong to the Tat/Ogre phylogenetic lineage of Ty3/gypsy elements [41,42], but are presented separately here as they were far more abundant than the rest of the families in this lineage (labeled as "Tat" in Fig 1B and S2 Table). The other two lineages of Ty3/gypsy, i.e. Chromovirus and Athila, as well as all Ty1/copia lineages known from plants [43,44] were also detected. Ty1/copia elements were generally less abundant in all genomes, with only Maximus/SIRE elements reaching the abundance of some of the Ty3/gypsy lineages (Fig 1B). Interestingly the amount of Angela elements in both *Pisum* species was much higher compared with the rest of the *Fabeae* species analysed (they represented 104–135 Mbp/1C in *Pisum* but only 0.7–34.3 Mbp/1C in other species, Table A in S2 Table).

Other groups of mobile elements detected included non-LTR retrotransposons (LINEs, SINEs), pararetrovirus sequences, five superfamilies of TIR-containing DNA transposons and Helitrons. However, only two DNA transposon superfamilies, *CACTA* and *Mutator*, were found in quantities greater than 10 Mbp/1C (and reaching up to 1.9% of some genomes).

In some species, relatively large amounts of satellite repeats were identified and these showed considerable sequence diversity. The highest absolute amounts of satellite DNA were found in *V*. *faba* (with 935 Mbp/1C; 6.97% of genome) and *L*. *sativus* (with 699 Mbp/1C; 10.7% of genome). Although less amplified, satellite repeats also made up a significant fraction of the genome in several species with small genomes like *V*. *villosa* (12.3%; 250 Mbp/1C) and *V*. *sativa* (10.4%; 184 Mbp/1C). The sequence composition of satellite DNA varied between species, with most species containing over 10 different sequence families (the largest numbers of putative satellites, 31 and 51, were identified in *V*. *faba* and *V*. *peregrina*, respectively). However, usually just a single or a few satellite repeats were dominant in terms of their genomic abundance (S3 Table).

### Reproducibility of data

Although most of the results described above were based on analysing reads from a single sequencing run per species, we also performed experimental replicates for selected samples to assess whether there was any bias in repeat quantification due to experimental factors. In two species, *V*. *pannonica* and *V*. *faba*, complete replicates, including independent library preparations and sequencing, were performed, while the impact of repeating only the sequencing runs from the same libraries was tested in three other species (*V*. *sepium*, *L*. *vernus*, *L*. *sativus*). It was found that while the latter had only a minor effect on read quantities in individual clusters, the sequencing data from different libraries showed more variation, although the extent of this depended, in part, on the type of repeat analysed. For example, when the quantities of different types of abundant genomic repeats were compared, the highest variability between datasets was found for clusters of satellite DNA, which in some cases showed up to four-fold variation (1.7–2.0 on average). In contrast, differences in read numbers for all groups of mobile elements were only up to two-fold between replicates (1.2–1.3 on average). Nevertheless, the variation was mostly eliminated when repeat quantities were calculated for whole groups of repeats by summing the read counts from all corresponding clusters (S2 Fig).

### Contribution of various groups of repeats to genome size diversification

Correlations between the amounts of different types of repetitive DNA with genome size variation in *Fabeae* were tested using absolute amounts (Mbp/1Cx) of repeats estimated for individual species (Table A in S2 Table). As expected, a strong positive correlation was found (R^2^ = 0.996, p < 2.20e^-16^) when data for all repeats were combined, and together they accounted for 85% of the genome size differences between species (Table 2). When individual repeat types were analysed separately, the strongest correlation was found for Ogre elements (R^2^ = 0.847, p = 4.99e^-10^), which, due to their high abundance, were directly responsible for about 57% of the genome size differences between species. The remaining groups of Ty3/gypsy elements, several groups of Ty1/copia elements and satellite DNA also showed significant positive correlations, but their contributions to genome size differences were much smaller compared to Ogres (Table 2).

**Table 2:** Correlation of repeat amounts with genome size variation in *Fabeae* and contribution of individual repeats to the genome size differences between species.

Repeat	Correlation to genome size		Abundance in analysed genomes [Mb/1Cx]		Average contribution to pairwise differences in genome sizes [%]
	R^2^	P-value	min.	max.	
Ogre	0.847	4.99e^-10^	398	7285	57.30
Ivana	0.510	0.00013	6	109	0.57
Maximus/SIRE	0.501	0.00016	74	908	4.80
Satellite	0.449	0.00047	6	935	2.40
Tat	0.314	0.00539	24	342	1.51
Athila	0.215	0.02590	20	332	1.64
Chromovirus	0.204	0.03070	71	1012	6.17
MITE	0.203	0.03090	0	4	-0.01
CACTA	0.202	0.03160	2	91	0.54
Tork	0.132	0.08780	1	52	0.25
rDNA	0.117	0.11000	6	110	0.37
All repeats	0.996	<2.2e^-16^	1053	11103	85.06

### Evolutionary dynamics of individual repeat types

**Fig 2:**
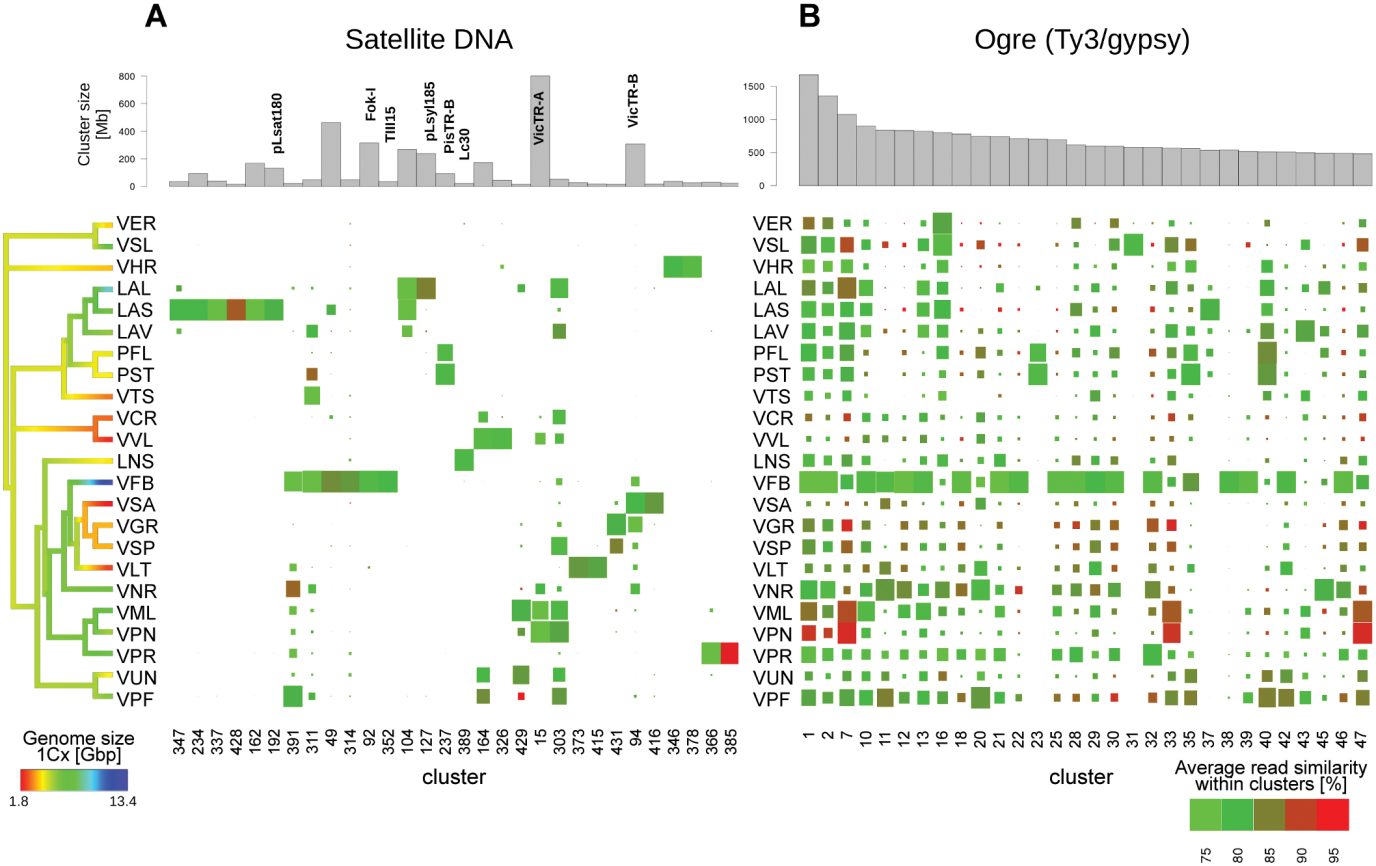
Phylogenetic distribution, abundance and sequence similarity between the thirty largest clusters representing satellite repeats (*A*) and Ogre retrotransposons (*B*) from the comparative clustering analysis. Bar plots at the top show cluster sizes (total length of all reads in Mbp) and rectangle areas below display the relative proportions of reads from individual species within each cluster. Previously described families of satellite repeats are marked with their names on panel *A*. The colours of the rectangles indicate the average pairwise similarities of read sequences.

To identify repeat variants (families) shared by multiple species and investigate their fate during *Fabeae* evolution we performed comparative repeat analysis by simultaneously clustering reads from all species. This approach resulted in clusters representing presumably orthologous repeat families from different species grouped together due to their high sequence similarity, and it allowed their quantification based on the number of reads from respective species. To provide equal sensitivity for all species, the number of reads analysed for each species was proportional to its genome size and corresponded to 0.01x genome coverage (Table 1, column "Clust. 0.01x"). In total 12.3 million reads were subjected to analysis, resulting in 9.97 million reads forming clusters of various sizes, with the 583 largest clusters (containing at least 0.005% of analysed reads) representing 71.3% of the analysed sequences. Fig 2A shows a graphical representation of part of the analysis for the 30 largest clusters of satellite DNA. These were found to be the least conserved class of repeats in the *Fabeae* genomes analysed. Such an analysis showed that the majority of satellite clusters were made up of reads from either a single species (e.g. all reads in CL234, 337 and 428 come from *Lathyrus sativus*) or just a few species (e.g. CL347 which contains reads from just *L*. *vernus*, *L*. *sativus* and *L*. *latifolius*), and suggests that satellite repeats in *Fabeae* are largely restricted to a limited number of species that are usually closely related.

In contrast, the comparative clustering analysis of most LTR-retrotransposons showed that most clusters contained reads from the majority or even all species, as shown for the 30 largest clusters of Ogre sequences (Fig 2B). Such results suggest that most LTR-retrotransposon repeat families are widely distributed across *Fabeae* although at varying abundances that are usually proportional to a species’ genome size (e.g., *V*. *faba* Ogre reads were the most abundant in most clusters).

An analysis of the average pairwise similarities between reads from individual species revealed that Ogre sequences were more homogeneous in some species (85–95% similarity), compared with others (75–85% similarity) suggesting recent amplification. This was most clearly seen for *V*. *pannonica* and *V*. *melanops* (clusters CL1, 7, 33 and 47), and interestingly also for some species with small genomes, like *V*. *grandiflora* and *V*. *cracca* (Fig 2B).

**Fig 3:**
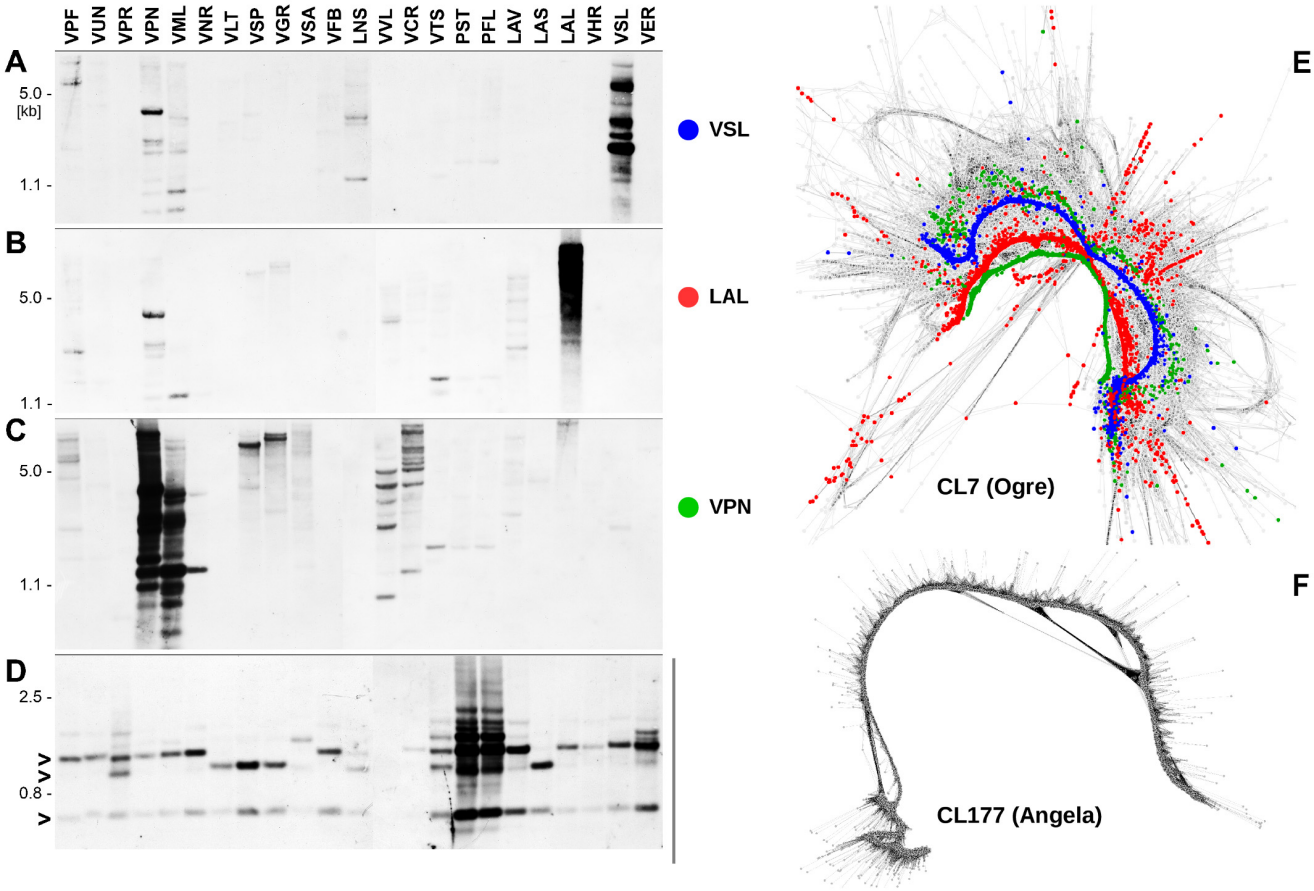
Southern blot detection of selected Ogre and Angela sequences in the genomes of *Fabeae* species. The blots were prepared from equal amounts of genomic DNA of each species digested with *Ssp*I and hybridized to probes corresponding to sequence variants of Ogre derived from *Vicia sylvatica* (*A*), *Lathyrus latifolius* (*B*) and *Vicia pannonica* (*C*). These variants are evident as narrow parallel paths on a graph representation [31] of cluster CL7 (*E*) where reads from these species are highlighted by blue, red and green colours, respectively (reads of all other species are in grey). For the Angela element cluster CL177, reads of all species (grey dots) generated a narrow linear graph due to their high sequence similarities (*F*). The corresponding probe detected several conserved bands (arrows) on the Southern blot (*D*).

Additional, more subtle differences representing sequence variants were distinguishable only by a detailed graph-based analysis of individual clusters. An example of this variability, typical for Ogre sequences is provided in Fig 3E, which shows a graphical representation of cluster CL7 corresponding to the RT/RH domains of an Ogre element (the proportion of reads from each species in this cluster is shown in Fig 2B). Multiple parallel paths were distinguishable on the graph, corresponding to sequence variants specific for a single or several species. Three of these *in silico* reconstructed sequences, representing Ogre variants from *V*. *pannonica*, *V*. *sylvatica* and *L*. *latifolius* are highlighted in different colors on Fig 3E and their existence *in vivo* was confirmed by their PCR-amplification from the respective genomes and sequencing. Similarities between the amplified fragments and the predicted sequences were >95%, and similarities between the variants from the three species were 73–77%. When the sequenced clones were used as probes for Southern hybridizations to *Fabeae* genomic DNAs, they generated signals preferentially in their species of origin (Fig 3A–3C), suggesting that the diversification of Ogre sequences accompanied their amplification within individual species or phylogenetic lineages of *Fabeae*.

In contrast to Ogre elements, sequence variability was much less evident in clusters of other retrotransposons and was almost absent in the Angela lineage of Ty1/copia elements. Here, despite the presence of reads from all analysed species, there were usually no parallel paths in the graphs representing species-specific sequence variants. This is clearly illustrated in Fig 3F, which shows the graph of cluster CL177 representing the PROT-INT-RT/RH domains of Angela elements. The probe derived from this region was cloned from the *P*. *sativum* genome but it had 88–97% similarity to corresponding element sequences from all other species. In addition, the probe revealed a number of conserved restriction fragments on the Southern blot which were present in most or all *Fabeae* species, thus confirming the extraordinary sequence conservation of this element (Fig 3D). This experiment also confirmed the amplification of Angela elements in both *Pisum* species analysed, as originally revealed by the quantification of repeats in individual species (Fig 1B, S2 Table).

**Fig 4:**
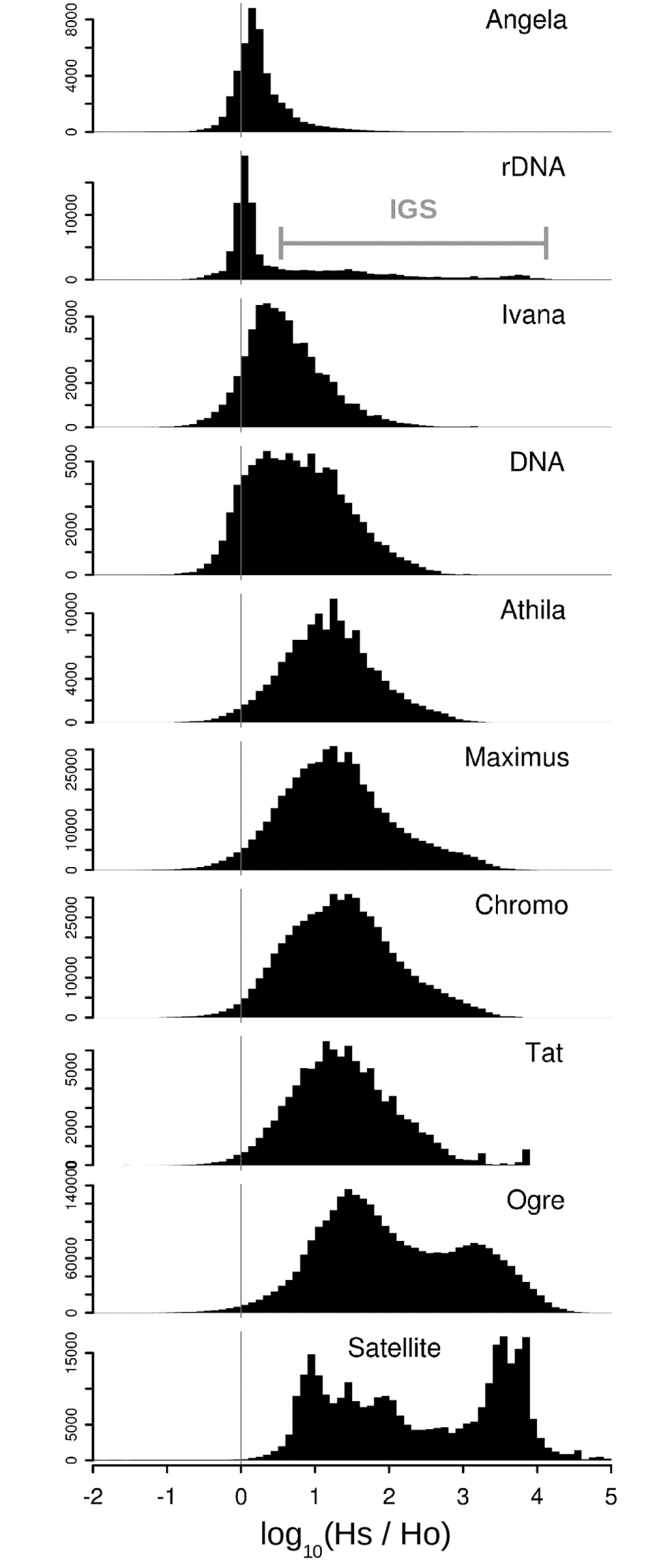
Sequence conservation of repeats between *Fabeae* species. The ratio *Hs*/*Ho* was calculated for each read within individual groups of repeats, where *Hs* was the frequency of similarity hits to reads from the same species and *Ho* was the frequency of hits to reads from all other species. The histograms show the distribution of *Hs*/*Ho* ratios for different repeats, with numbers of reads plotted along the y-axis. The *Hs*/*Ho* ratios are close to 1 (0 on the log scale) for highly conserved sequences whereas larger values correspond to sequence divergence, resulting in higher frequencies of hits within than between species (for example, a value of 2 on the x-axis corresponds to reads producing 100-fold more intra-specific than inter-specific hits).

Prompted by the results described above, we designed an additional assay to investigate global variability of individual repeat types across the *Fabeae* genomes. It was based on pooling reads for each species and repeat type into a separate dataset, evaluating their pairwise (intra-specific) similarities and comparing them to the similarities of reads from the same repeat in all other species. The resulting ratios of intra- versus inter-specific similarity hits (*Hs*/*H*o ratios) are plotted in Fig 4, and reveal that there are considerable differences in the histogram profiles between repeat types and lineages. In theory, a repeat whose sequences are highly conserved in all species will produce similarity hits with about the same frequency when comparing reads within and between species (the frequencies were normalized to a total number of reads representing the repeat in a given species, thus they were independent of varying copy numbers of the repeat in analysed species). Such a high degree of interspecific sequence conservation was confirmed for Angela elements, generating a narrow peak close to the zero value on the log scale, and indicating that ratios of intra- (*Hs*) to inter- (*Ho*) specific hit frequencies were close to one. A similar pattern was obtained for ribosomal DNA (rDNA) sequences, where the histogram profile comprised a peak reflecting high sequence conservation of the rRNA genes and a broad right hand tail generated by the divergent intergenic spacer sequences (IGS). The opposite pattern, indicative of highly divergent sequences, was found for satellite repeats. An interesting bi-modal histogram was obtained for Ogre elements, suggesting there were two fractions of sequences differing in the extent of their conservation between species (Fig 4). Further investigation where repeats were compared only within groups of phylogenetically closely related species revealed a simplification of the histograms shown in Fig 4, and their shift towards zero (i.e. 1:1 ratio), indicating higher repeat similarities within more closely related species than with all *Fabeae* (S3 Fig).

### Proportions of solo-LTRs as an indicator of genome shrinkage

Compared to genomic expansions, usually governed by amplification of repetitive elements, the corresponding signatures of genome downsizing are in principle much harder to detect in NGS data. Repeat elimination is considered to be mainly driven by sequence excision following recombination within or between repeat copies [22] or by non-specific DNA loss associated with double-strand break repair [6]. However, currently there are no tools available which allow these phenomena to be analysed using short read sequences generated by NGS approaches. Nevertheless, taking advantage of our deep annotation of repeats in *Fabeae*, we attempted to investigate at least one of these mechanisms—the elimination of LTR-retrotransposon sequences by ectopic recombination between the LTRs of the same element. This process leaves solo-LTRs in place of the original full-length elements and thus the proportion of solo-LTRs to intact element copies can be used to indicate the extent of element removal from the genome by ectopic recombination. We have developed a novel bioinformatic approach to estimate this proportion by quantifying the number of reads containing the junction between the 3' end of the LTR (LTR_3'end) and the internal retrotransposon region (i.e. 5' UTR, starting with the primer binding site) and the reads containing just the LTR_3'end alone, and hence representing an insertion site of the element (see S4 Fig for detailed explanation). The calculation results in *Rsf* value, providing the estimated ratio of solo-LTRs to full-length elements in the genome. An *Rsf* value of 0 indicates that all elements are full-length whereas the occurrence of solo-LTRs is reflected by an excess of LTR_3'end insertion sites and hence an *Rsf* value >0.

Performing this analysis for all types of abundant LTR-retrotransposons did not reveal any profound differences in the ratios of solo-LTRs to full-length elements either between species or types of LTR retrotransposons (*Rsf* values ranged from 0 to 2 for most elements; Table 3). Nevertheless, it was evident from the graphical representation in Fig 1B, that smaller amounts of Ogre sequences were often accompanied by higher proportions of solo-LTRs, although there were exceptions. For example, the ratio of solo-LTRs to complete Ogre elements was higher than might be expected for the relatively large genome of *L*. *latifolius* (9.98 Gbp/1C, *Rsf* = 1.6). In addition, there were several groups of closely related species whose genomes showed consistent differences in the proportions of solo-LTRs. These included the two analysed *Pisum* species, where the one with the slightly smaller genome (*P*. *sativum*) contained over two-fold higher proportion of solo-LTRs for all major retrotransposons (especially Ty/gypsy elements) compared with *P*. *fulvum*. Similarly, the species with the smallest genome, *V*. *sativa*, had a relatively high proportion of solo-LTRs (*Rsf* = 0.9–2.0) compared with its related species *V*. *grandiflora* (*Rsf* = 0.2–0.9) and *V*. *sepium* (*Rsf* = 0.0–1.5) which have genomes that are over twice as big (highlighted in Table 3). Such results suggest that this mechanism of repeat removal may well contribute to genome size evolution at least in some of the analysed species.

**Table 3:** Estimated ratios of solo-LTRs to complete elements (*Rsf*).

Species	1Cx	Ty3/gypsy				Ty1/copia			
	[Gbp]	Ogre	Chromo.	Tat	Athila	Max/Sire	Ivana	Tork	Angela
VER	4.06	1.0	0.2	0.5	0.4	1.5	*-*	0.6	*-*
VSL	6.98	0.6	0.7	0.4	0.4	0.2	*-*	0.4	*-*
VHR	3.88	1.2	0.7	0.5	0.7	0.8	*-*	*-*	*-*
LAL	9.98	1.6	0.8	0.7	1.0	0.4	2.0	2.0	*-*
LAS	6.52	0.6	0.3	0.7	0.5	0.6	*-*	*-*	*-*
LAV	5.91	1.0	0.0	0.0	0.2	0.5	0.3	0.2	1.4
**PFL**	**4.69**	**0.6**	**0.0**	**0.4**	**0.8**	**0.1**	**0.8**	***-***	**0.9**
**PST**	**4.36**	**1.8**	**0.5**	**1.0**	**1.9**	**0.5**	**0.9**	***-***	**1.0**
VTS	3.05	1.9	1.3	0.5	0.7	1.2	*-*	1.0	2.6
VCR	2.90	1.1	0.3	0.3	0.3	0.3	0.8	0.7	*-*
VVL	2.04	1.0	0.8	0.5	0.1	0.4	*-*	1.2	*-*
LNS	4.29	0.6	0.4	0.2	1.0	0.7	*-*	*-*	*-*
VFB	13.41	0.7	0.5	0.5	1.3	1.6	4.4	*-*	1.1
**VSA**	**1.77**	**1.6**	**1.4**	**0.9**	**1.6**	**1.4**	**2.0**	**2.3**	***-***
**VGR**	**3.78**	**0.3**	**0.3**	**0.3**	**0.6**	**0.2**	**0.9**	***-***	***-***
**VSP**	**3.74**	**1.5**	**0.0**	**0.2**	**1.0**	**0.3**	**0.7**	***-***	***-***
VLT	2.43	0.9	0.8	0.6	1.0	1.4	2.0	1.8	*-*
VNR	6.69	0.5	0.5	0.4	0.5	0.4	*-*	*-*	*-*
VML	8.07	0.5	0.4	0.4	0.5	0.5	0.7	1.7	*-*
VPN	5.73	0.5	0.3	0.5	0.5	0.5	0.9	0.9	*-*
VPR	8.45	0.7	0.4	0.7	0.7	1.2	*-*	*-*	*-*
VUN	4.37	1.0	0.5	0.5	0.2	0.9	0.5	1.1	*-*
VPF	6.15	0.5	0.1	0.2	0.2	0.6	0.4	0.3	*-*

## Discussion

### The challenge of comparative repeat analysis

The high proportions and diversity of repetitive DNA sequences in plant genomes raise significant methodological challenges for their detailed analysis and annotation, leaving a significant fraction of repeats poorly characterized even in many extensively studied model genomes. These difficulties become even more evident when conducting a comparative analysis of repeats between multiple species. Nevertheless, such studies are crucial for elucidating their evolutionary dynamics and understanding their impact on plant genome size, organization, expression and evolution. Although several studies have been conducted using assembled genome sequences [45] and shotgun sequencing approaches of small insert genomic libraries [46–48], it has been the advent of NGS that has opened up the possibility of gaining deep insights into the repeat composition of species across the diversity of genome sizes encountered in plants [49,50]. Despite these new opportunities, however, most studies using NGS data have focused on one or a few species [26] and thus comparative repeat analyses of multiple species still remain scarce [14,16,17]. In the present work, we have taken advantage of the combined potential of NGS and clustering-based repeat identification pipeline to demonstrate that such an approach can be scaled up to characterize over twenty genomes simultaneously, and with sufficient analysis depth, to enable all types of moderately to highly repeated sequences to be identified and thoroughly analysed.

Since such methodology is relatively novel, it is essential to evaluate its accuracy, both in terms of its ability to faithfully represent the nucleotide sequences of repeats present in the genome, as well as provide reliable quantitative information about their relative abundance. By cloning/sequencing, Southern and *in situ* hybridization, it has already been demonstrated that repetitive elements reconstructed from read clustering data accurately represent the repeats present in the genome [16,19,51,52]. In addition, the visualization of populations of repeats in the genome using their cluster graph representations has even led to the identification of otherwise hardly detectable structures such as centromeric satellites with extremely long monomers [52]. Further confirmation of the methodology is provided in the present work, as cloned probes matched predicted sequences in the genome with over 95% accuracy, and Southern blot experiments were also in agreement with the clustering data (Fig 3).

In addition to accurately identifying the different types of repeats present, there are several methodological issues in the Illumina sequencing workflow that may cause biased quantifications of certain genomic sequences. For example, since genomic DNA has to be fragmented and size-fractionated prior to preparing the sequencing library, increased fragility or resistance to fragmentation of certain sequences compared with the bulk of genomic DNA may cause their under-representation in the selected fraction of fragments. In addition, significant deviations from an average GC content of the genome (especially a high % GC) are known to interfere with PCR-based library amplification, causing a depletion of the corresponding templates [53]. In relation to repetitive DNA, these factors are most likely to impact the quantification of satellite repeats as they are composed of long arrays of relatively short monomers and often contain runs of A/T homopolymers [54], thus forming regions of low sequence complexity differing from the rest of the genome. Indeed, in our control replica experiments conducted for this study, we observed that clusters containing satellite repeats showed greater variation in read numbers compared with the clusters of transposable elements (S2 Fig). Nevertheless, the observed differences in the amount of satellite repeat families between species spanned several orders of magnitude, and thus abundance estimates for individual satellites were not significantly affected by experimental error acting on a scale of just a few-fold variation. In addition, a comparison of the results presented here (Fig 2A and S2 Table) with previously published data on genomic abundances of the *Vicia* VicTR-A and VicTR-B satellites estimated by membrane hybridization experiments [55] did not show any significant discrepancies. For the quantification of the mobile elements which make up the majority of *Fabeae* repeats, the variability in cluster sizes between the control replica experiments was smaller compared to satellites (S2 Fig) and this variation could be further reduced by quantifying individual groups of transposons by summing read numbers from all clusters representing a given group. Further support is also provided by the good agreement between estimates of Ogre proportions in the *V*. *pannonica* genome determined experimentally (38%, [21]) and here by computational analysis of NGS data (44%, Table B in S2 Table). Taken together, it can be concluded that the precision of our assays does not significantly compromise the key results of this study.

### Ogre elements as the major force driving the genome size evolution in *Fabeae*

Recent decades of genome investigation have led to the recognition that LTR-retrotransposons comprise the main component of the repeated fraction of the genome in most plant species studied so far (see reviews by [4,26,56]). However, as judged from the contrasting reports describing genomes of different species being dominated by a single or multiple families of either, Ty3/gypsy and Ty1/copia elements [14,15,20,45,46], there appears to be no simple pattern in LTR-retrotransposon evolution that explains the genome size diversity encountered in plants. In part, this might be due to our limited and fragmentary knowledge derived from data obtained from single species or small groups of related taxa, and it emphasizes the need for broader sampling to bridge these gaps. In addition, the impact of contrasting population sizes and different ecological and mating strategies of plant species on their repeat composition are also likely to be significant, yet such studies are still relatively rare [57–59].

The data presented here confirmed the crucial role of LTR-retrotransposons in governing genome size evolution in plants. In the particular case of *Fabeae*, the analyses have provided robust evidence for the pivotal role that Ogre elements have played in genome size evolution in this tribe. A similar impact of a single lineage of LTR-retrotransposons has also been reported, for example, in the genus *Gossypium* [46], but to our knowledge our study is the first case where this phenomenon has been documented across such a broad taxonomic sampling comprising many genera. Ogres are probably an evolutionary young lineage of Ty3/gypsy elements, being present in multiple eudicot families but up to now not detected in any non-eudicot angiosperm. Although they are exceptional in their large size (up to 25 kb) and organization of their coding regions [42,60], it is unlikely that these features alone are the key to their successful proliferation, as the same elements are generally less abundant outside the *Fabeae*. For example, in the closely related legume genus of *Trifolium*, which is sister to the *Fabeae* tribe, Ogres account for just 2.4% of the *T*. *pratense* genome (1C = 418 Mbp), and instead Maximus (Ty1/copia) and Chromovirus (Ty3/gypsy) are the most abundant lineages of LTR-retrotransposons [61]. Based on the ancestral genome size reconstruction and the repeat similarity analyses, it is hypothesized that Ogre amplification has accompanied speciation in *Fabeae*. However it is not yet clear if the amplification of these repeats has occurred continuously over time or in multiple bursts. Certainly, episodes of rapid proliferation followed by silencing and elimination have been reported for LTR-retrotransposons in several other plant species [45] and these "TE-thrusts" were proposed to coincide with evolutionary radiation and speciation events [20,62]. The broad and bimodal distribution of sequence similarity profiles of Ogres (Fig 4 and S3 Fig) suggest the presence of at least two distinct subpopulations of Ogre elements which differ in their sequence conservation, although it has yet to be determined whether these different subpopulations reflect amplification events that differ in age. Currently, the dating of LTR-retrotransposon insertions can most reliably be achieved by quantifying the sequence divergence of the LTRs within individual element copies. However, such information is not available when analysing short sequence read data. Indeed, as already noted above, the employed methodology also has limited power with respect to characterizing the various processes causing repeat elimination, which is an important part of our understanding of genome size evolution. Nevertheless, the solo-LTR estimation method introduced here has provided the first step towards addressing this problem, and the results obtained are in the range of solo-LTR amounts reported from analysing whole genome assemblies [45], providing support for the reliability of our approach. In addition, the increased solo-LTR proportions of Ogres (and other elements) were mostly consistent with presumed cases of genome shrinkage in some *Fabeae* species.

### Contrasting patterns of evolution between different groups of repetitive elements

The comparative analysis of repeats in *Fabeae* has revealed profound differences between the various types of repeats with respect to both, their abundance and sequence conservation. The most evolutionary dynamic repeats were shown to be satellite DNAs, resulting in highly divergent sequence families which were mostly restricted to a single or just a few closely related species, and were absent from the rest of the *Fabeae*. This is in line with the high turnover rates observed for satellite DNAs in other taxa [63]. However, we have also encountered an unexpected diversity of satellite DNAs in some species, where over twenty families differing in monomer sizes and nucleotide sequences have been identified (S3 Table). Although only a few of them were usually amplified to high copy numbers, such repeats are clearly genuine satellites, with well homogenized sequences presumably organized into long arrays, as previously shown for *Pisum sativum* [37]. Since previous studies have typically reported just a few satellite repeat families within a species [54,63], it has yet to be shown whether this unexpected diversity of satellite DNAs found here is specific for *Fabeae* or just reflects the higher sensitivity of our analytical approach.

As discussed above, the Ogre elements were shown to be divergent with regards to their sequences in different species (e.g. Fig 3E), which probably arose from the amplification of different families or sequence variants within individual species. Such higher sequence variability distinguished all lineages of Ty3/gypsy from most Ty1/copia elements (Fig 4). Indeed, we have noticed high sequence conservation in other Ty1/copia elements by examining patterns of the corresponding cluster graphs in several unrelated species outside the *Fabeae* (data not shown), suggesting this might be a general feature of Ty1/copia elements. This is further supported by the observation that members of the Angela lineage (which was shown to be the most conserved LTR-retrotransposon in *Fabeae*) retain partial similarities in nucleotide sequences between elements present in plant families as distant as legumes (*Fabaceae*) and grasses (*Poaceae*) [64]. Similarly, another Ty1/copia element, PARTC, was recently reported to be conserved across gymnosperms [65]. Our preliminary experiments using *in situ* hybridization of an Angela probe to *P*. *sativum* chromosomes produced uniformly dispersed signals, suggesting that these elements do not occupy specific chromosome regions which could eventually explain their slower mutation rates (data not shown). Overall, it seems clear that similar experiments and corresponding genomic studies across a large number of diverse taxa are needed to explore the extent to which our observations are typical across plants or specific to just the *Fabeae* and they will be conducted in the future to investigate these interesting phenomena in more detail.

## Materials and Methods

### Plant material and genome size estimation

Seeds of most *Vicia* species were obtained from the seed bank of the Leibniz Institute of Plant Genetics and Crop Plant Research (IPK), Gatersleben, Germany. Commercial varieties of *V*. *pannonica*, *V*. *faba* and *Pisum sativum* were obtained from Osiva Boršov, Czech Republic, *V*. *sativa* from the Agricultural Research Institute Kroměříž, Czech Republic, *Lens culinaris* from the Nohel garden, Dobříš, Czech Republic and *V*. *narbonensis* (ICARDA 14) was provided by A. M. Torrres (IFAPA Cordoba, Spain). Seeds of *Lathyrus sativus* and *L*. *latifolius* were purchased from Fratelli Ingegnoli S.p.A., Milano, Italy (cat.no. 455) and SEMO Smržice, Czech Republic (acc.no. 1-0040-68867-01), respectively. *Lathryus vernus* was collected from a wild population at Vidov, Czech Republic (GPS 48°55'17.401"N, 14°29'44.158"E). *Pisum fulvum* accession (ICARDA IG64207) was provided by Petr Smýkal, Palacký University, Olomouc, Czech Republic. Herbarium vouchers were archived for all investigated species at the Laboratory of Molecular Cytogenetics, Biology Centre CAS, České Budějovice, Czech Republic.

Nuclear DNA content was estimated using flow cytometry according to [66]. Intact leaf tissues of a sample and reference standard were chopped together in a glass Petri dish containing 500 μl Otto I solution (0.1 M citric acid, 0.5% v/v Tween 20) using a sharp razor blade. The crude suspension was filtered through a 50 μm nylon mesh. Nuclei were then pelleted (300 *g*, 3 min) and resuspended in 300 μl Otto I solution. After 15 min incubation at room temperature, 900 μl Otto II solution (0.4 M Na_2_HPO_4_) [67] supplemented with 50 μg/ml RNase and 50 μg/ml propidium iodide, were added. Samples were analysed using a Partec PAS flow cytometer (Partec GmbH, Münster, Germany) equipped with a high-pressure mercury arc lamp. At least 5,000 nuclei were analysed per sample. Three plants of each species were analysed and each plant was measured three times on three different days. The reference standards used in this study were soybean (*Glycine max* L. cv. Polanka, 2C = 2.5 pg DNA) [68], maize (*Zea mays* cv. C-777, 2C = 5.43 pg DNA) [69] and pea (*Pisum sativum* cv. Ctirad, 2C = 9.09 pg DNA) [70]. Nuclear DNA content was estimated using the formula: sample 2C nuclear DNA content [pg] = sample G_1_ peak mean × standard 2C DNA content [pg] / standard G_1_ peak mean. Mean nuclear DNA content (2C) was then calculated for each plant. DNA amounts in picograms were converted to the number of base pairs using the conversion factor 1 pg DNA = 0.978 x10^9^ bp [71].

### Genomic DNA sequencing and repeat characterization from NGS reads

Genomic DNA used for sequencing was extracted from isolated leaf nuclei as described [19] except for *V*. *villosa* where total genomic DNA was used instead. Shotgun sequencing of randomly sheared DNA was performed by Elim Biopharmaceuticals, Hayward, USA (*V*. *sativa*, *V*. *pannonica*, *V*. *faba*, *P*. *sativum* and *P*. *fulvum*) and GATC Biotech, Konstanz, Germany (all other species, including additional runs of *V*. *pannonica* and *V*. *faba*), employing an Illumina platform and protocol generating 100 nt paired-end reads from ~200–400 bp fragment libraries. Sequencing data for *P*. *sativum* was generated earlier using the same technology [37].

Repeat identification by similarity-based clustering of Illumina reads was performed using local installation of the *RepeatExplorer* pipeline [32] which was run on a Debian Linux server with 32 CPU cores and 64 GB RAM. The pipeline employs graph representation of read similarities to identify clusters of frequently overlapping reads representing various repetitive elements or their parts [31]. In addition, it provides information about repeat quantities (estimated from the number of reads in a cluster), information about cluster connections via paired-end reads used to identify repeats split between multiple clusters, and outputs from BLASTn and BLASTx [72] similarity searches to our custom databases of repetitive elements and repeat-encoded conserved protein domains that aid in repeat annotation. This information was combined and used for final manual annotation and quantification of repeats from all clusters making up at least 0.01% of investigated genomes. The analysis was performed on each species separately, using the maximum number of randomly sampled reads that could be processed (see Table 1 for details). [31]

In addition, a simultaneous comparative repeat analysis of all genomes was performed by clustering a combined dataset made by pooling reads representing 0.01x genome coverage of each species (Table 1). This coverage was chosen as a compromise providing good sensitivity of the analysis while requiring moderate run time and computational resources. Following the analysis and annotation of clusters making up at least 0.005% of analysed reads, proportions of reads in each cluster from individual species were determined by parsing read names where species of origin were encoded by specific tags. Calculations of pair-wise read similarities within clusters were performed separately for reads from each species represented in the cluster. Sequence similarities were detected using BLASTn with word size set to 7 (-W 7) to increase search sensitivity. The program output was parsed to calculate average similarities for each group of reads, taking into account only pair-wise hits longer than 50 bp and performing appropriate corrections for positions of hits within reads (adjusting similarity values for read overlaps in case the hits did not reach read ends). All analyses which were not part of the *RepeatExplorer* pipeline output were performed using custom scripts in BioPerl (http://www.bioperl.org) and R (http://www.r-project.org/). Graph representations of individual clusters were investigated using the SeqGrapheR program [31].

### Sequence conservation of repeats between *Fabeae* species

Repeat conservation between species was calculated by parsing the read similarities reported by *RepeatExplorer* for the comparative clustering analysis of reads representing 0.01x genome coverage of each species. The pipeline employs the mgblast program [73] to find similarity hits satisfying the specified threshold of 90% similarity over a region of at least 55 bp. Lists of reads representing different groups of repeats were assembled from clusters with the same annotations (e.g. all reads from clusters annotated as "satellite" were grouped together). The *Hs/Ho* ratios were then calculated for each read within these groups, where *Hs* is the frequency of hits to reads from the same species and *Ho* is the frequency of hits to reads from all other species. The frequencies were obtained by dividing the number of reads with similarities by the total number of reads within the groups, thus providing normalization for varying amounts of repeats in different species.

### Proportions of solo-LTRs

The procedure used to estimate ratios of solo-LTRs to full-length LTR-retrotransposons is schematically depicted in supplementary S4 Fig. A set of BioPerl scripts was developed to identify putative 3' borders of long terminal repeats (LTR_3'end) and adjacent 5' untranslated regions (5'UTRs) of LTR-retrotransposons. As an input, the scripts used contig assembly (ACE) files generated by assembling reads within each cluster (provided as part of the *RepeatExplorer* output). These assemblies were scanned for regions of abrupt increases in the proportion of masked read sequences which are indicative of insertion sites of mobile elements into unrelated genomic sequences. These sites were considered as potential LTR_3'end/5'UTR regions only if there was (i) a conserved LTR terminal dinucleotide (TG/CA) at the proper position and also (ii) a region of similarity to tRNA sequence representing a retrotransposon primer binding site (PBS) detected within the potential 5'UTR. The scripts were applied to clustering data from individual species and potential LTR_3'end/5'UTR boundaries were confirmed by manual examination. Thirty-nucleotide sequence tags were then extracted from the validated LTR_3'end and 5'UTR sequences from positions directly adjacent to their boundaries and these tags were used to create BLAST databases. The databases were used to detect corresponding regions in individual sequence reads using BLASTn with "-W 7 -e 0.01" parameters. The search output was parsed to quantify reads with significant hits (at least 90% similarity over 27 bp) either to both LTR_3'end and 5'UTR tags in the proper position and orientation, or to the LTR_3'end tags alone (these were counted only in cases when there was at least a 30 bp region left between the LTR_3'end tag and the end of the read and this region did not have similarity to the 5'UTR tag). The ratios of solo-LTRs to full-length elements were then calculated as *Rsf* = (*Lx*—*LU*)/*LU*, where *LU* is the number of reads containing a LTR_3'end/5'UTR region and *Lx* is the number of reads containing a LTR_3'end alone.

### Phylogenetic analysis and ancestral genome size reconstruction

Nuclear (ITS) and chloroplast loci (*trnS-C* and *matK*) were used in this study for phylogenetic reconstructions using corresponding sequences assembled from the NGS datasets. Nucleotide sequences were aligned manually using BioEdit v. 7.0.5.3 [74]. Phylogenetic reconstructions using Bayesian inference (BI) were carried out with MrBayes v. 3 [75]. Partitions were made for the three loci and subsequently analysed both individually and as a concatenated dataset; the most appropriate nucleotide substitution models for each partition were chosen under the Akaike information criterion (AIC) with MrModeltest v. 2 [76]. For each analysis four Markov chains were run simultaneously for 1 x 10^6^ generations and sampled every 100 generations. The MCMC sampling was considered sufficient as the effective sample size (ESS) was > 200 in each case after evaluating in Tracer v.1.5 (http://tree.bio.ed.ac.uk/software/tracer/). Data from the first 2000 samples were discarded in each analysis, and the remaining trees were used to construct 50% majority-rule consensus tree. Posterior probabilities (PP) of nodes were calculated from the pooled samples.

A sample of 1000 post-burn in trees from the initial BI was reformatted with BayesTrees v.1.3 (http://www.evolution.reading.ac.uk/BayesTrees.html) and then used in reconstructing the ancestral genome sizes in BayesTraits v.2 (http://www.evolution.rdg.ac.uk/BayesTraits.html). Monoploid (1Cx-values) genome size data were log transformed in order to ensure a normal distribution (Kolmogorov-Smirnov test, P = 0.213) of the values. The best model for analysis of continuously varying characters (random walk *vs*. directional walk) was selected by performing BayesFactor tests using the logarithm of the harmonic mean estimated after conducting five separate MCMC runs with the following prior settings: sampling every 1000 generations, 100 million iterations, burn-in of 10 million iterations. The scaling parameters δ, κ and λ were estimated. All parameter values were inspected with Tracer v.1.5 to ensure they were stationary. The random walk model was favoured in most of the preliminary runs which, together with the posterior distribution of the scaling parameters generated, was used to set the model for the second phase of the analysis in which we estimated the genome size of internal nodes by using the add MRCA command.

### Southern blot hybridization

To prepare probes for Southern hybridizations, selected variants of Ogre sequences from cluster CL7 of the comparative analysis were PCR-amplified from genomic DNA of *V*. *sylvatica* (primers FComp_7c1027-F: 5'-AAA GTG TAC CTT TGG TGT CAG-3' and FComp_7c1027-R: 5'-TTC TTC AAC TGC AAA GAT ATG AGC-3') and L. latifolius (FComp_7c2717-F: 5'-TGA TAT CGT GAT ACC AAG GTT TGT C-3' and FComp_7c2717-R: 5'-CAG GGA AAC TCT TAG GGT TCA TC-3'). Alternatively, an Ogre sequence variant representative for V. pannonica was amplified from the genomic cosmid clone VPcosC6 (GenBank accession AY936172) using the primers FComp_7c2240-F (5'-GAC ATG ATC GCC AAG TCC AG-3') and FComp_7c2240-R (5'-GGC TGC AAA CAC ATA AGC TG-3'). The probe for Angela elements (spanning clusters CL177 and CL241) was amplified from P. sativum genomic DNA using the primers FJ434420-F (5'-GAG GAA CCT CCT AGT TTT GCA C-3') and FJ434420-R (5'-ATC CCA CGC TCT TTC AGA TG-3'). All fragments amplified from genomic DNAs were cloned and sequenced. The sequence-verified clones were then used to prepare hybridization probes by labelling them with Biotin-16-dUTP (Roche) during PCR amplification.

Southern blots were prepared using 1.5 μg aliquots of genomic DNAs digested with *Ssp*I, resolved on 1% agarose gels and blotted to Hybond N+ membranes (Amersham) by capillary transfer. The blots were hybridized with 10 ng/ml of biotin-labelled probe in 5x SSC, 5 x Denhardt's solution, 1% (w/v) SDS, 100 μg/ml salmon sperm DNA at 68°C overnight. Stringent post-hybridization washes were performed at 60°C in 2 x 15 min in 0.2x SSC/0.1% SDS and 1 x 15 min in 0.1x SSC/0.1% SDS. Hybridization signals were detected using Ultra SNAP Detection Kit (Vector Laboratories) and DuoLux Chemiluminescent/Fluorescent Substrate and visualized by exposure to an X-ray film.

## Supporting Information

S1 Fig Fifty percent majority-rule consensus tree from Bayesian inference (ITS, trnS-C, matK) showing the phylogenetic relationships of investigated *Fabeae* species. (PDF)

S2 Fig Comparison of read quantities from major groups of repeats obtained from two repetitions of sequencing runs. (PDF)

S3 Fig Sequence conservation of repeats within groups of closely related species. (PDF)

S4 Fig Principle of solo-LTR detection from NGS reads. (PDF)

S1 Table Genome size estimations of *Fabeae* species. (PDF)

S2 Table Total length and proportions of individual repeat types in investigated genomes. (PDF)

S3 Table Numbers of satellite DNA families identified in repeat clustering data. (PDF)
