# Supplementary material for: In Depth Characterization of Repetitive DNA in 23 Plant Genomes Reveals Sources of Genome Size Variation in the Legume Tribe Fabeae
Source: PLoS One. 2015 Nov 25;10(11):e0143424. doi: 10.1371/journal.pone.0143424 (PMC4659654; doi:10.1371/journal.pone.0143424)
Supplement: S1 Fig — (PDF) [file pone.0143424.s001.pdf]

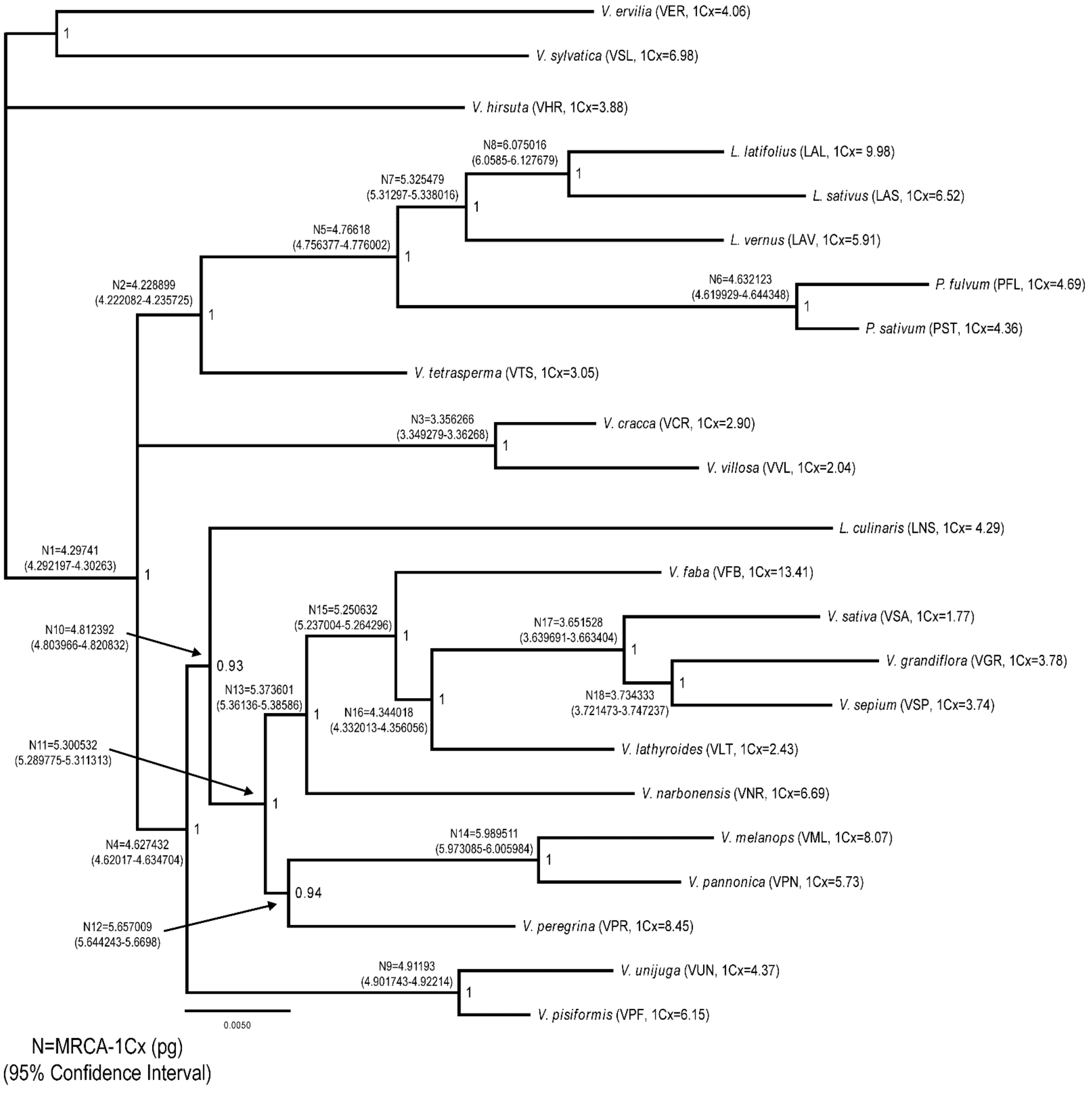

Supplementary Fig. S1 - Fifty percent majority-rule consensus tree from Bayesian inference (ITS, trnS-C, matK) showing the phylogenetic relationships of the 23 investigated *Fabeae* species. Posterior probabilities are indicated for each node. The ancestral genome sizes reconstructed (1Cx-values [pg]) for the most recent common ancestor (MRCA) of each clade are depicted together with the 95% confidence interval in brackets.
