# Supplementary material for: In Depth Characterization of Repetitive DNA in 23 Plant Genomes Reveals Sources of Genome Size Variation in the Legume Tribe Fabeae
Source: PLoS One. 2015 Nov 25;10(11):e0143424. doi: 10.1371/journal.pone.0143424 (PMC4659654; doi:10.1371/journal.pone.0143424)
Supplement: S2 Fig — (PDF) [file pone.0143424.s002.pdf]

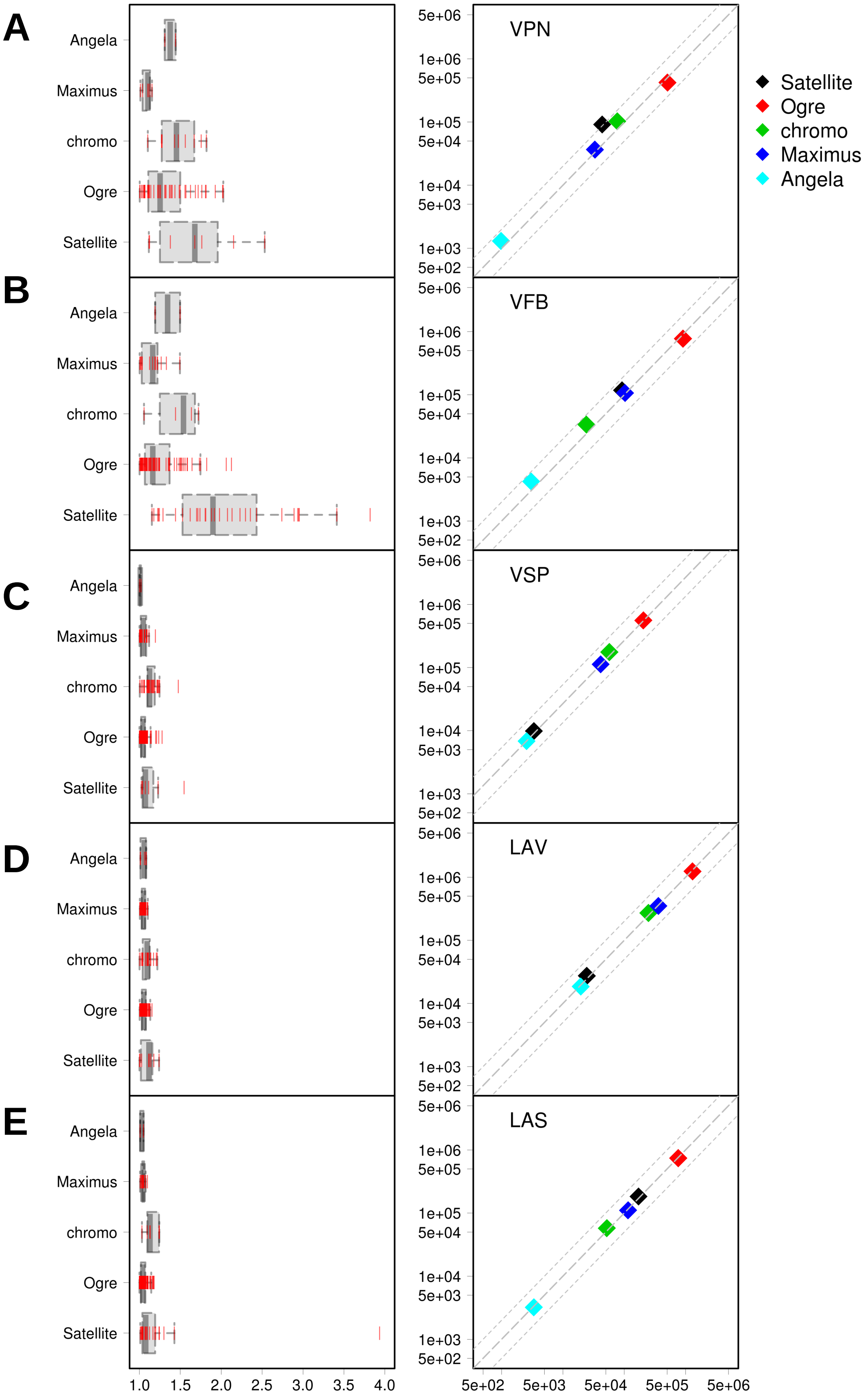

Supplementary Fig. S2. Comparison of read quantities from major groups of repeats obtained from two repetitions of sequencing runs. In *V. pannonica* (A) and *V. faba* (B), the two runs were performed using two independently prepared sequencing libraries, whereas in *V. sepium* (C), *L. vernus* (D) and *L. sativus* (E) the repeated runs were performed from the same template libraries. *Left panels* show fold differences in read numbers between the two repetitions in individual clusters (red lines) and box-plots (gray) mark median, first and thirds quartiles and maximum and minimum of these values. *Right panels* provide scatter plots of total read quantities summed for repeat groups and compared between the repetitions. Dotted lines above and below the diagonals mark two-fold differences in repeat quantities.
