# Supplementary material for: In Depth Characterization of Repetitive DNA in 23 Plant Genomes Reveals Sources of Genome Size Variation in the Legume Tribe Fabeae
Source: PLoS One. 2015 Nov 25;10(11):e0143424. doi: 10.1371/journal.pone.0143424 (PMC4659654; doi:10.1371/journal.pone.0143424)
Supplement: S3 Fig — (PDF) [file pone.0143424.s003.pdf]

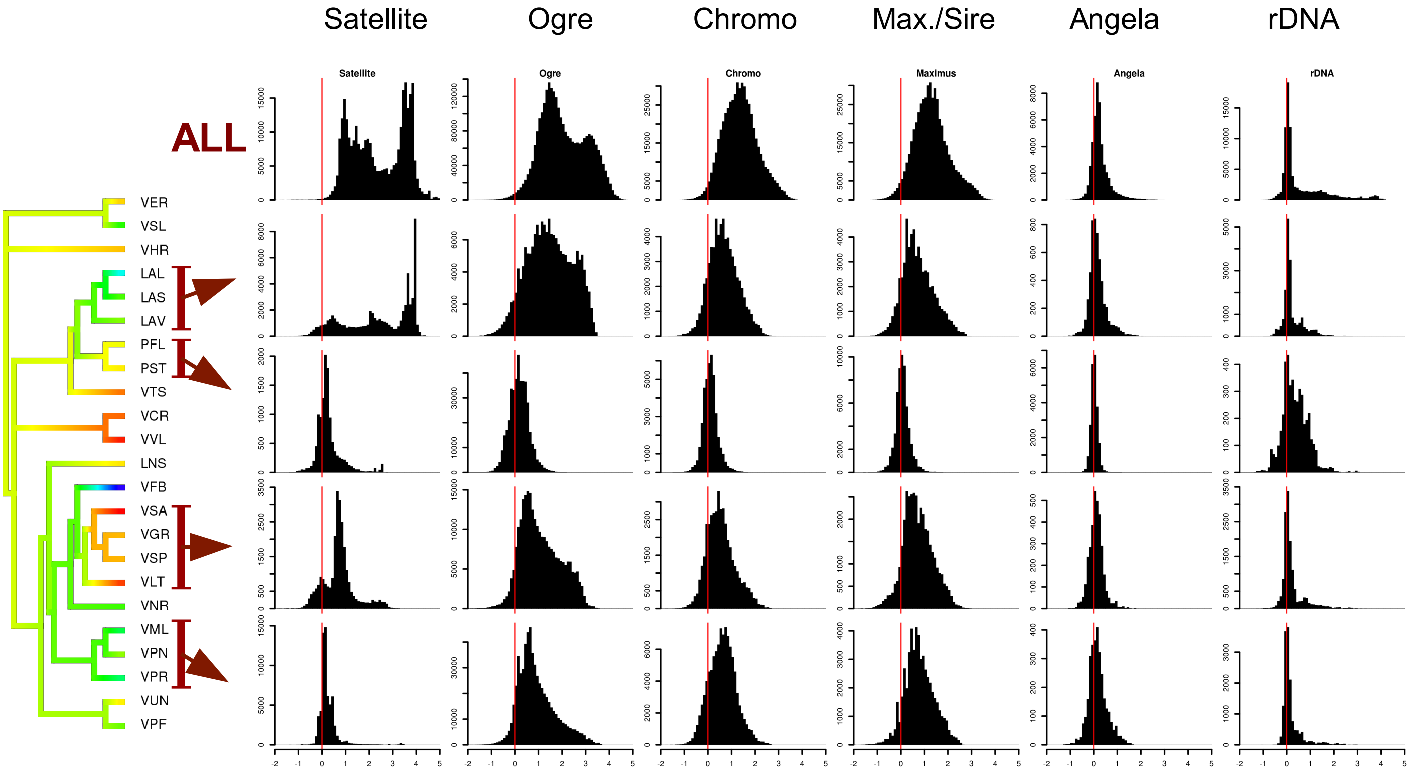

Supplementary Fig. S3. Sequence conservation of repeats within groups of closely related species. The Hs/Ho ratios were calculated either for all *Fabeae* (ALL), or only for groups of two to four species as marked by the brown dimension bars on the phylogenetic tree. For explanation of the histograms see Fig. 4.
