# Supplementary material for: In Depth Characterization of Repetitive DNA in 23 Plant Genomes Reveals Sources of Genome Size Variation in the Legume Tribe Fabeae
Source: PLoS One. 2015 Nov 25;10(11):e0143424. doi: 10.1371/journal.pone.0143424 (PMC4659654; doi:10.1371/journal.pone.0143424)
Supplement: S4 Fig — (PDF) [file pone.0143424.s004.pdf]

Principle of solo-LTR detection from NGS reads

Step 1. Identification of LTR_3'end/5'UTR junctions from read assemblies.
Contig assemblies are scanned for abrupt increases in the proportion of masked read regions (gray color) representing heterogeneous sequences of the element insertion sites. The primer binding site (PBS, detected by similarity to tRNA) and LTR end consensus dinucleotide TG/CA have to be present in the correct orientation.

Assembled reads marked with red rectangles represent LTR/5'UTR junctions, green rectangles show the reads derived from LTR end/insertion site regions.

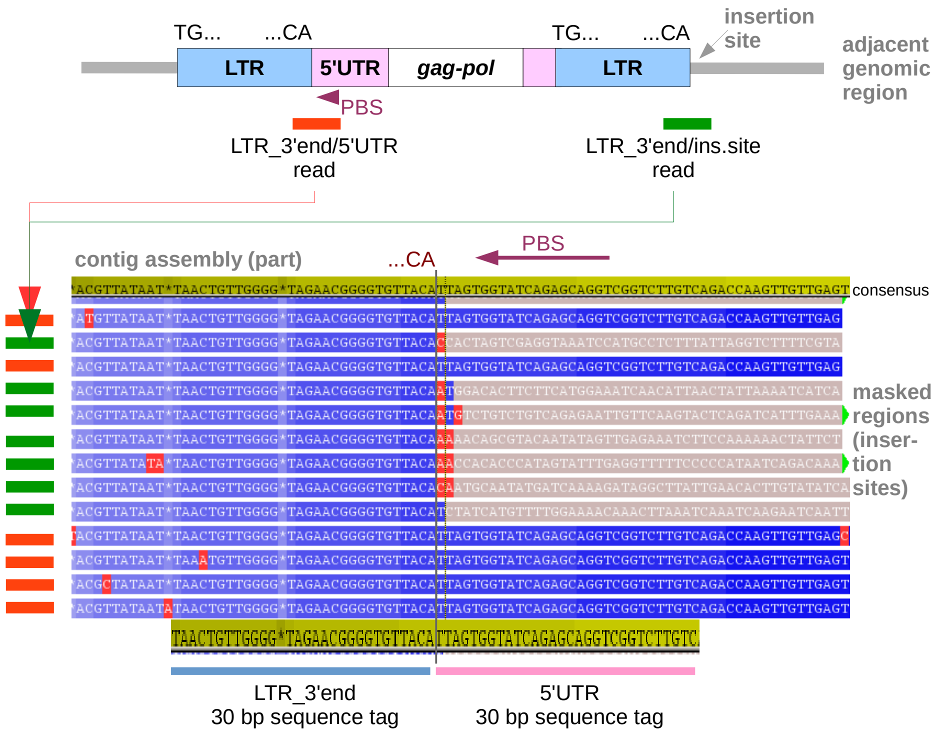

Step 2. Extraction of 30 bp sequence tags and creating BLAST databases.
Tags are collected from all identified LTR retrotransposons and their classification is encoded into tag IDs.

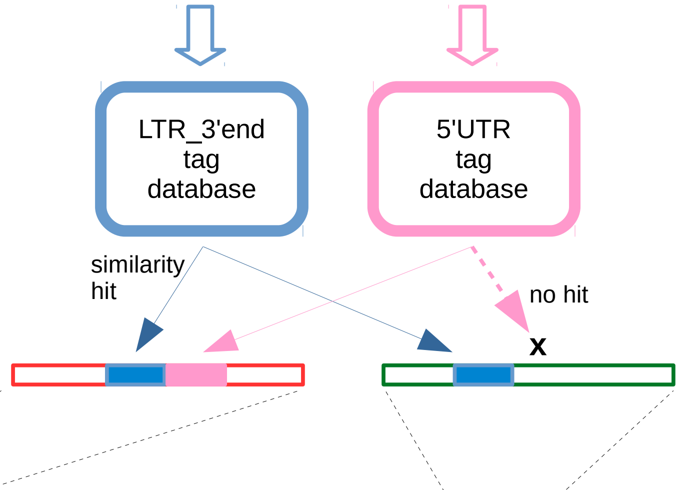

Step 3. BLAST of all read sequences to the tag databases.
Reads with hits to both, the LTR3'end and 5'UTR tags represent LTR/5'UTR junctions (red circles). Reads from the LTR/insertion site (green circles) lack hits to 5'UTR.

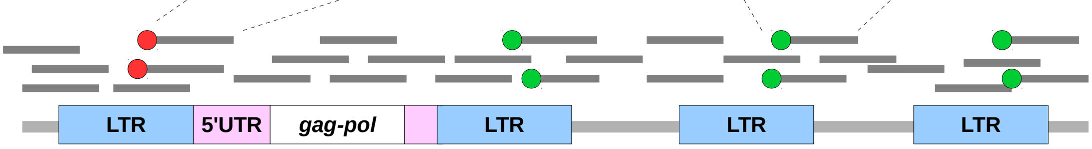

Step 4. Parsing and quantifying BLAST hits.
In the example provided here, the ratio of solo-LTRs to full-length elements in the genome is 2:1. This will result in corresponding quantities of reads representing LTR_3'end/5'UTR junctions (*LU*) and LTR_3'end only (*Lx*). The ratio *Rsf* is then calculated using the formula.

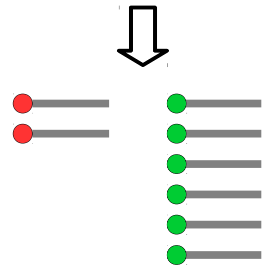

LU *Lx*

$$Rsf = (Lx - LU) / LU$$

$$Rsf = (6 - 2) / 2 = \mathbf{2}$$
