# Supplementary material for: In Depth Characterization of Repetitive DNA in 23 Plant Genomes Reveals Sources of Genome Size Variation in the Legume Tribe Fabeae
Source: PLoS One. 2015 Nov 25;10(11):e0143424. doi: 10.1371/journal.pone.0143424 (PMC4659654; doi:10.1371/journal.pone.0143424)
Supplement: S1 Table — (PDF) [file pone.0143424.s005.pdf]

Supplementary Table S1. Genome size estimations of *Fabeae* species

**Species**	**Code**	**Accession**		**Chrom.**	**Genome size**				
		**source**	**code**	**number [2n]**	**2n [pg]**	**SD**	**std.**	**1C [Gbp]**	**1Cx [Gbp]**
Vicia									
V. sativa 'Ebena'	**VSA**	commercial		12	3.627	0.025	ZM	**1.77**	**1.77**
V. villosa	**VVL**	IPK	VIC876	14	4.164	0.056	GM	**2.04**	**2.04**
V. lathyroides L.	**VLT**	IPK	VIC874	12	4.960	0.019	ZM	**2.43**	**2.43**
V. cracca L. var. *cracca*	**VCR**	IPK	VIC71	28	11.848	0.055	PS	**5.79**	**2.90**
V. tetrasperma (L.) Schreb.	**VTS**	IPK	VIC726	14	6.245	0.015	GM	**3.05**	**3.05**
V. sepium L.	**VSP**	IPK	VIC55	14	7.656	0.047	ZM	**3.74**	**3.74**
V. grandiflora	**VGR**	IPK	VIC741	14	7.732	0.087	ZM	**3.78**	**3.78**
V. hirsuta (L.) S.F.Gray	**VHR**	IPK	VIC728	14	7.925	0.049	ZM	**3.88**	**3.88**
V. ervilia (L.) Willd.	**VER**	IPK	ERV52	14	8.297	0.067	ZM	**4.06**	**4.06**
V. unijuga A.Br.	**VUN**	IPK	VIC78	12	8.932	0.055	ZM	**4.37**	**4.37**
V. pannonica 'Dětěnická panonská'	**VPN**	commercial		12	11.720	0.069	PS	**5.73**	**5.73**
V. pisiformis L.	**VPF**	IPK	VIC36	12	12.585	0.018	PS	**6.15**	**6.15**
V. narbonensis	**VNR**	ICARDA	14	14	13.675	0.064	PS	**6.69**	**6.69**
V. sylvatica L.	**VSL**	IPK	VIC63	14	14.280	0.063	PS	**6.98**	**6.98**
V. melanops Sibth. et Sm. var. *melanops*	**VML**	IPK	VIC474	10	16.495	0.036	PS	**8.07**	**8.07**
V. peregrina L.	**VPR**	IPK	VIC765	14	17.273	0.043	PS	**8.45**	**8.45**
V. faba 'Merkur'	**VFB**	commercial		12	27.423	0.214	PS	**13.41**	**13.41**
Lens				14					
L. culinaris 'Eston'	**LNS**	commercial		14	8.771	0.027	ZM	**4.29**	**4.29**
Lathyrus									
L. vernus (L.) Bernh.	**LAV**	natural population		14	12.091	0.040	PS	**5.91**	**5.91**
L. sativus L.	**LAS**	commercial		14	13.343	0.094	PS	**6.52**	**6.52**
L. latifolius L.	**LAL**	commercial		14	20.404	0.092	PS	**9.98**	**9.98**
Pisum									
P. sativum 'Terno'	**PST**	commercial		14	8.921	0.020	ZM	**4.36**	**4.36**
P. fulvum	**PFL**	ICARDA	IG64207	14	9.587	0.020	ZM	**4.69**	**4.69**

Genome size standards (column “std.”): ZM, *Zea mays*; GM, *Glycine max*; PS, *Pisum sativum*
