# Supplementary material for: In Depth Characterization of Repetitive DNA in 23 Plant Genomes Reveals Sources of Genome Size Variation in the Legume Tribe Fabeae
Source: PLoS One. 2015 Nov 25;10(11):e0143424. doi: 10.1371/journal.pone.0143424 (PMC4659654; doi:10.1371/journal.pone.0143424)
Supplement: S2 Table — (PDF) [file pone.0143424.s006.pdf]

A – Total length [Mbp] of individual repeat types per monoploid genome

species:	VSA	VVL	VLT	VCR	VTS	VSP	VGR	VHR	VER	VUN	VPN	VPF	VNR	VSL	VML	VPR	VFB	LNS	LAV	LAS	LAL	PST	PFL
1Cx[Gbp]:	1.77	2.04	2.43	2.90	3.05	3.74	3.78	3.88	4.06	4.37	5.73	6.15	6.69	6.98	8.07	8.45	13.41	4.29	5.91	6.52	9.98	4.36	4.69
LTR/gypsy																							
**Ogre**	397.8	512.7	965.5	736.7	1025.5	1538.7	2124.5	1761.2	1883.7	1186.3	2538.8	2929.2	4328.8	3930.2	4450.8	3263.8	7284.8	1532.6	2282.4	2963.7	3063.5	2079.1	2697.5
**Tat**	29.3	49.5	75.9	133.0	50.8	128.1	91.8	97.1	135.4	148.1	88.9	144.9	110.2	84.5	141.5	236.3	253.4	117.0	341.5	33.1	161.0	24.1	30.6
**Chrom**	80.4	70.7	150.2	396.0	115.0	470.0	232.0	365.0	545.2	562.3	491.4	512.2	339.7	988.9	610.9	488.1	251.2	602.2	466.8	216.9	1012.0	182.5	259.5
**Athila**	20.4	50.0	74.8	105.1	133.3	78.1	89.2	110.0	100.6	173.0	85.6	185.4	108.6	75.4	162.6	218.0	145.6	69.3	332.1	202.7	188.4	158.2	173.2
LTR/copia																							
**Max.**	73.6	209.0	90.4	345.5	332.4	310.6	250.5	371.5	290.9	483.3	191.1	498.9	250.7	264.5	297.5	755.9	907.6	484.2	638.7	446.8	469.5	415.6	274.2
**Ivana**	19.0	5.8	28.1	26.2	35.9	40.6	19.1	17.9	13.2	52.5	47.4	55.8	21.9	32.7	47.5	34.1	109.0	14.5	69.6	19.0	72.1	61.5	39.6
**Angela**	3.2	0.7	6.8	2.2	12.5	18.9	11.5	3.7	16.0	16.1	6.2	10.6	18.9	8.9	8.0	24.9	31.5	6.7	34.3	13.0	29.8	135.1	103.6
**Tork**	8.3	8.2	17.8	24.7	16.6	8.0	0.6	9.0	29.6	22.0	29.5	35.6	9.4	36.8	21.4	19.9	10.1	10.6	30.7	21.3	52.3	12.0	5.7
**Bianka**	1.6	3.5	5.1	4.5	2.0	0.8	1.2	2.2	3.1	12.2	3.7	5.0	1.4	1.6	0.0	11.7	0.0	0.0	2.7	0.0	10.2	0.0	0.0
**TAR**	0.0	6.6	10.6	7.9	3.5	0.5	0.6	3.8	2.4	5.1	7.9	9.4	7.3	0.0	12.1	4.6	7.5	1.5	3.7	4.5	5.4	11.5	4.3
**AleII**	0.8	1.0	3.2	2.8	5.3	0.0	1.6	1.9	4.1	4.6	0.0	2.0	3.9	1.7	1.0	2.4	0.0	0.5	3.4	1.0	0.0	7.2	1.8
**AleI**	0.0	0.0	0.0	0.0	0.0	0.0	0.0	0.0	0.0	0.0	0.0	0.0	0.0	2.6	0.0	0.0	0.0	0.0	0.0	0.0	0.0	0.4	1.1
**TRIM**	0.2	0.0	0.9	1.0	1.4	1.0	0.0	0.5	0.7	0.7	0.0	1.0	0.0	2.0	2.3	0.0	0.0	0.8	0.8	1.8	0.0	16.6	11.5
**LTR unclas**	123.5	16.1	168.1	59.5	87.3	70.4	93.0	39.3	20.1	96.8	294.1	137.2	80.7	223.6	116.9	519.8	189.5	108.7	159.8	329.8	1436.1	34.9	6.6
**LINE**	0.0	0.0	0.0	1.7	2.2	0.5	0.0	0.9	0.0	2.1	0.0	1.6	0.0	0.0	1.0	0.0	0.0	7.3	5.0	0.0	0.0	0.0	0.0
**SINE**	0.0	0.3	0.0	0.0	0.0	0.0	0.0	0.0	0.0	0.0	0.0	0.0	0.0	0.0	0.0	0.0	0.0	0.0	0.0	0.0	0.0	0.0	0.0
**PARA**	0.0	0.0	1.3	2.0	3.4	0.0	0.0	1.5	0.5	4.2	0.0	5.3	0.0	19.2	0.0	1.9	0.0	0.5	4.9	0.0	5.5	0.0	1.2
DNA																							
**CACTA**	16.1	5.9	9.4	26.2	56.9	8.6	4.9	23.0	65.9	83.7	20.6	90.8	20.1	47.5	50.4	56.1	81.7	72.0	51.3	1.6	47.4	34.6	25.2
**Mutator**	6.7	12.3	11.1	31.3	51.7	22.9	11.6	19.2	18.9	34.0	5.0	10.6	8.4	20.0	12.1	47.1	35.1	25.0	29.6	2.0	24.0	44.0	49.8
**hAT**	0.0	1.8	0.3	4.6	2.8	0.0	0.0	0.0	1.3	7.9	0.0	0.0	0.0	0.0	0.0	1.0	0.0	0.5	2.5	0.7	2.9	3.3	1.4
**PIF/Har.**	0.0	0.7	0.0	1.5	0.8	0.0	0.0	0.0	0.0	2.5	0.0	0.0	0.0	0.0	0.0	1.2	0.0	0.0	0.0	0.0	0.0	0.0	0.0
**Tc1/Mar.**	0.0	0.0	0.0	0.0	0.0	0.0	0.0	0.0	0.0	0.0	0.0	0.0	0.0	0.0	1.2	0.0	0.0	0.0	0.0	0.0	0.0	0.0	0.0
**MITE**	1.1	3.6	1.8	1.3	0.0	0.4	2.7	0.0	0.0	2.7	0.6	0.7	0.0	0.0	0.8	0.0	0.0	0.0	0.8	0.0	0.0	0.0	0.0
**unclass.**	0.0	17.1	0.3	2.4	0.0	0.0	8.5	0.8	4.8	0.0	0.0	0.0	0.0	0.0	6.8	0.0	4.8	3.5	0.0	17.0	26.1	0.0	5.0
**Helitron**	0.3	0.0	1.0	7.8	6.6	0.0	0.0	0.0	0.0	8.4	0.0	0.0	0.0	0.0	0.0	7.8	1.9	2.7	3.5	0.0	0.0	1.6	0.0
**Satellite**	184.4	250.0	77.0	49.6	45.5	26.7	101.8	109.2	22.5	45.5	369.5	90.7	140.2	6.4	293.7	183.0	935.1	49.0	46.7	699.4	364.9	92.0	72.6
**Tandem**	0.0	0.0	5.1	1.5	15.8	9.1	9.9	44.5	0.0	15.4	0.0	20.1	7.2	1.2	6.0	174.8	367.8	24.2	24.2	56.2	11.4	1.7	1.3
**rDNA**	5.5	10.5	42.5	25.8	29.5	54.8	58.4	55.1	34.2	22.5	48.2	33.3	25.3	54.5	64.5	64.6	45.1	49.7	73.0	110.2	31.2	11.7	40.8
**unclass.**	80.7	46.2	45.1	87.3	170.6	91.3	38.0	100.4	44.3	233.3	100.1	263.3	146.9	178.9	60.6	525.3	441.3	317.6	164.7	155.4	519.6	149.6	65.6
**% genome**	**59.5**	**62.9**	**73.8**	**72.0**	**72.4**	**77.0**	**83.4**	**80.9**	**79.7**	**73.8**	**75.5**	**82.0**	**84.2**	**85.7**	**78.9**	**78.6**	**82.8**	**81.6**	**80.8**	**81.2**	**75.5**	**79.8**	**82.6**
**% annot.**	**92.3**	**96.4**	**97.5**	**95.8**	**92.3**	**96.8**	**98.8**	**96.8**	**98.6**	**92.8**	**97.7**	**94.8**	**97.4**	**97.0**	**99.0**	**92.1**	**96.0**	**90.9**	**96.5**	**97.1**	**93.1**	**95.7**	**98.3**
**% gen.ann.**	**54.9**	**60.6**	**71.9**	**69.0**	**66.8**	**74.6**	**82.4**	**78.3**	**78.6**	**68.5**	**73.8**	**77.7**	**82.0**	**83.1**	**78.2**	**72.4**	**79.5**	**74.2**	**78.0**	**78.8**	**70.3**	**76.3**	**81.2**

B – Genome proportions [%] of repeats

species:	VSA	VVL	VLT	VCR	VTS	VSP	VGR	VHR	VER	VUN	VPN	VPF	VNR	VSL	VML	VPR	VFB	LNS	LAV	LAS	LAL	PST	PFL
1Cx[Gbp]:	1.77	2.04	2.43	2.9	3.05	3.74	3.78	3.88	4.06	4.37	5.73	6.15	6.69	6.98	8.07	8.45	13.41	4.29	5.91	6.52	9.98	4.36	4.69
**LTR/gypsy**																							
**Ogre**	22.48	25.13	39.73	25.40	33.62	41.14	56.20	45.39	46.40	27.15	44.31	47.63	64.71	56.31	55.15	38.63	54.32	35.72	38.62	45.46	30.70	47.69	57.52
**Tat**	1.66	2.43	3.12	4.59	1.66	3.42	2.43	2.50	3.34	3.39	1.55	2.36	1.65	1.21	1.75	2.80	1.89	2.73	5.78	0.51	1.61	0.55	0.65
**Chrom**	4.54	3.47	6.18	13.65	3.77	12.57	6.14	9.41	13.43	12.87	8.58	8.33	5.08	14.17	7.57	5.78	1.87	14.04	7.90	3.33	10.14	4.19	5.53
**Athila**	1.15	2.45	3.08	3.62	4.37	2.09	2.36	2.84	2.48	3.96	1.49	3.02	1.62	1.08	2.02	2.58	1.09	1.62	5.62	3.11	1.89	3.63	3.69
**LTR/copia**																							
**Max.**	4.16	10.24	3.72	11.91	10.90	8.31	6.63	9.57	7.16	11.06	3.34	8.11	3.75	3.79	3.69	8.95	6.77	11.29	10.81	6.85	4.70	9.53	5.85
**Ivana**	1.07	0.29	1.16	0.90	1.18	1.09	0.51	0.46	0.33	1.20	0.83	0.91	0.33	0.47	0.59	0.40	0.81	0.34	1.18	0.29	0.72	1.41	0.85
**Angela**	0.18	0.03	0.28	0.08	0.41	0.51	0.30	0.10	0.39	0.37	0.11	0.17	0.28	0.13	0.10	0.29	0.23	0.16	0.58	0.20	0.30	3.10	2.21
**Tork**	0.47	0.40	0.73	0.85	0.54	0.21	0.02	0.23	0.73	0.50	0.51	0.58	0.14	0.53	0.27	0.24	0.08	0.25	0.52	0.33	0.52	0.28	0.12
**Bianka**	0.09	0.17	0.21	0.16	0.07	0.02	0.03	0.06	0.08	0.28	0.06	0.08	0.02	0.02	0.00	0.14	0.00	0.00	0.05	0.00	0.10	0.00	0.00
**TAR**	0.00	0.32	0.44	0.27	0.11	0.01	0.02	0.10	0.06	0.12	0.14	0.15	0.11	0.00	0.15	0.05	0.06	0.03	0.06	0.07	0.05	0.26	0.09
**AleII**	0.05	0.05	0.13	0.10	0.17	0.00	0.04	0.05	0.10	0.11	0.00	0.03	0.06	0.02	0.01	0.03	0.00	0.01	0.06	0.02	0.00	0.17	0.04
**AleI**	0.00	0.00	0.00	0.00	0.00	0.00	0.00	0.00	0.00	0.00	0.00	0.00	0.00	0.04	0.00	0.00	0.00	0.00	0.00	0.00	0.00	0.01	0.02
**TRIM**	0.01	0.00	0.04	0.03	0.05	0.03	0.00	0.01	0.02	0.02	0.00	0.02	0.00	0.03	0.03	0.00	0.00	0.02	0.01	0.03	0.00	0.38	0.25
**LTR unclas**	6.98	0.79	6.92	2.05	2.86	1.88	2.46	1.01	0.49	2.22	5.13	2.23	1.21	3.20	1.45	6.15	1.41	2.53	2.70	5.06	14.39	0.80	0.14
**LINE**	0.00	0.00	0.00	0.06	0.07	0.01	0.00	0.02	0.00	0.05	0.00	0.03	0.00	0.00	0.01	0.00	0.00	0.17	0.08	0.00	0.00	0.00	0.00
**SINE**	0.00	0.01	0.00	0.00	0.00	0.00	0.00	0.00	0.00	0.00	0.00	0.00	0.00	0.00	0.00	0.00	0.00	0.00	0.00	0.00	0.00	0.00	0.00
**PARA**	0.00	0.00	0.05	0.07	0.11	0.00	0.00	0.04	0.01	0.10	0.00	0.09	0.00	0.28	0.00	0.02	0.00	0.01	0.08	0.00	0.06	0.00	0.03
**DNA**																							
**CACTA**	0.91	0.29	0.39	0.90	1.87	0.23	0.13	0.59	1.62	1.92	0.36	1.48	0.30	0.68	0.63	0.66	0.61	1.68	0.87	0.03	0.48	0.79	0.54
**Mutator**	0.38	0.61	0.46	1.08	1.70	0.61	0.31	0.50	0.47	0.78	0.09	0.17	0.13	0.29	0.15	0.56	0.26	0.58	0.50	0.03	0.24	1.01	1.06
**hAT**	0.00	0.09	0.01	0.16	0.09	0.00	0.00	0.00	0.03	0.18	0.00	0.00	0.00	0.00	0.00	0.01	0.00	0.01	0.04	0.01	0.03	0.08	0.03
**PIF/Har.**	0.00	0.04	0.00	0.05	0.03	0.00	0.00	0.00	0.00	0.06	0.00	0.00	0.00	0.00	0.00	0.01	0.00	0.00	0.00	0.00	0.00	0.00	0.00
**Tc1/Mar.**	0.00	0.00	0.00	0.00	0.00	0.00	0.00	0.00	0.00	0.00	0.00	0.00	0.00	0.00	0.02	0.00	0.00	0.00	0.00	0.00	0.00	0.00	0.00
**MITE**	0.06	0.18	0.07	0.04	0.00	0.01	0.07	0.00	0.00	0.06	0.01	0.01	0.00	0.00	0.01	0.00	0.00	0.00	0.01	0.00	0.00	0.00	0.00
**unclass.**	0.00	0.84	0.01	0.08	0.00	0.00	0.23	0.02	0.12	0.00	0.00	0.00	0.00	0.00	0.08	0.00	0.04	0.08	0.00	0.26	0.26	0.00	0.11
**Helitron**	0.02	0.00	0.04	0.27	0.22	0.00	0.00	0.00	0.00	0.19	0.00	0.00	0.00	0.00	0.00	0.09	0.01	0.06	0.06	0.00	0.00	0.04	0.00
**Satellite**	10.42	12.26	3.17	1.71	1.49	0.72	2.69	2.82	0.55	1.04	6.45	1.48	2.10	0.09	3.64	2.17	6.97	1.14	0.79	10.73	3.66	2.11	1.55
**Tandem**	0.00	0.00	0.21	0.05	0.52	0.24	0.26	1.15	0.00	0.35	0.00	0.33	0.11	0.02	0.07	2.07	2.74	0.56	0.41	0.86	0.11	0.04	0.03
**rDNA**	0.31	0.51	1.75	0.89	0.97	1.46	1.54	1.42	0.84	0.52	0.84	0.54	0.38	0.78	0.80	0.77	0.34	1.16	1.24	1.69	0.31	0.27	0.87
**unclass.**	4.56	2.26	1.86	3.01	5.59	2.44	1.01	2.59	1.09	5.34	1.75	4.28	2.20	2.56	0.75	6.22	3.29	7.40	2.79	2.38	5.21	3.43	1.40
**total**	59.5	62.9	73.8	72.0	72.4	77.0	83.4	80.9	79.7	73.8	75.5	82.0	84.2	85.7	78.9	78.6	82.8	81.6	80.8	81.2	75.5	79.8	82.6
**annotated**	54.9	60.6	71.9	69.0	66.8	74.6	82.4	78.3	78.6	68.5	73.8	77.7	82.0	83.1	78.2	72.4	79.5	74.2	78.0	78.8	70.3	76.3	81.2
