# Supplementary material for: In Depth Characterization of Repetitive DNA in 23 Plant Genomes Reveals Sources of Genome Size Variation in the Legume Tribe Fabeae
Source: PLoS One. 2015 Nov 25;10(11):e0143424. doi: 10.1371/journal.pone.0143424 (PMC4659654; doi:10.1371/journal.pone.0143424)
Supplement: S3 Table — (PDF) [file pone.0143424.s007.pdf]

Supplementary Table S3. Numbers of satellite DNA families identified in repeat clustering data

Code	Species	Genome proportion [a]			
		**Total**	**>1.0%**	**0.1-1.0%**	**<0.1%**
VER	*V. ervilia* (L.) Willd.	10	0	1	9
VSL	*V. sylvatica* L.	3	0	0	3
VHR	*V. hirsuta* (L.) S.F.Gray	14	0	9	5
LAL	*L. latifolius* L.	7	2	3	2
LAS	*L. sativus* L.	23	4	5	14
LAV	*L. vernus* (L.) Bernh.	8	0	1	7
PFL	*P. fulvum*	19	0	3	16
PST	*P. sativum* 'Terno'	15	1	4	10
VTS	*V. tetrasperma* (L.) Schreb.	15	0	3	12
VCR	*V. cracca L.* var. *cracca*	19	0	4	15
VVL	*V. villosa*	23	3	4	16
LNS	*L. culinaris* 'Eston'	23	0	1	22
VFB	*V. faba* 'Merkur'	31	2	6	23
VSA	*V. sativa* 'Ebena'	18	1	4	13
VGR	*V. grandiflora*	13	1	1	11
VSP	*V. sepium* L.	6	0	1	5
VLT	*V. lathyroides* L.	24	1	3	20
VNR	*V. narbonensis*	9	1	1	7
VML	*V. melanops Sibth. et Sm.* var. *melanops*	18	1	0	17
VPN	*V. pannonica* 'Dětěnická panonská'	7	1	1	5
VPR	*V. peregrina* L.	51	0	3	48
VUN	*V. unijuga* A.Br.	4	0	2	2
VPF	*V. pisiformis* L.	24	0	3	21
	**Total:**	**384**	**18**	**63**	**303**

[a] Putative satellite repeats identified in each species were ranked into three categories based on their estimated genomic abundance (expressed as genome percentage). The table gives numbers of different satellite repeat families in each category.
